# Melanocortin 1 Receptor Deficiency in Hematopoietic Cells Promotes the Expansion of Inflammatory Leukocytes in Atherosclerotic Mice

**DOI:** 10.3389/fimmu.2021.774013

**Published:** 2021-11-19

**Authors:** James J. Kadiri, Sina Tadayon, Keshav Thapa, Anni Suominen, Maija Hollmén, Petteri Rinne

**Affiliations:** ^1^ Research Centre for Integrative Physiology & Pharmacology, Institute of Biomedicine, University of Turku, Turku, Finland; ^2^ Drug Research Doctoral Programme (DRDP), University of Turku, Turku, Finland; ^3^ MediCity Research Laboratory, University of Turku, Turku, Finland; ^4^ Turku Center for Disease Modeling, University of Turku, Turku, Finland

**Keywords:** melanocortin 1 receptor, atherosclerosis, CD4^+^ T cell, inflammation, bone marrow transplantation, hematopoiesis, leukocytosis, leukocyte migration

## Abstract

Melanocortin receptor 1 (MC1-R) is expressed in leukocytes, where it mediates anti-inflammatory actions. We have previously observed that global deficiency of MC1-R signaling perturbs cholesterol homeostasis, increases arterial leukocyte accumulation and accelerates atherosclerosis in apolipoprotein E knockout (Apoe^-/-^) mice. Since various cell types besides leukocytes express MC1-R, we aimed at investigating the specific contribution of leukocyte MC1-R to the development of atherosclerosis. For this purpose, male Apoe^-/-^ mice were irradiated, received bone marrow from either female Apoe^-/-^ mice or MC1-R deficient Apoe^-/-^ mice (Apoe^-/-^ Mc1r^e/e^) and were analyzed for tissue leukocyte profiles and atherosclerotic plaque phenotype. Hematopoietic MC1-R deficiency significantly elevated total leukocyte counts in the blood, bone marrow and spleen, an effect that was amplified by feeding mice a cholesterol-rich diet. The increased leukocyte counts were largely attributable to expanded lymphocyte populations, particularly CD4^+^ T cells. Furthermore, the number of monocytes was elevated in Apoe^-/-^ Mc1r^e/e^ chimeric mice and it paralleled an increase in hematopoietic stem cell count in the bone marrow. Despite robust leukocytosis, atherosclerotic plaque size and composition as well as arterial leukocyte counts were unaffected by MC1-R deficiency. To address this discrepancy, we performed an *in vivo* homing assay and found that MC1-R deficient CD4^+^ T cells and monocytes were preferentially entering the spleen rather than homing in peri-aortic lymph nodes. This was mechanistically associated with compromised chemokine receptor 5 (CCR5)-dependent migration of CD4^+^ T cells and a defect in the recycling capacity of CCR5. Finally, our data demonstrate for the first time that CD4^+^ T cells also express MC1-R. In conclusion, MC1-R regulates hematopoietic stem cell proliferation and tissue leukocyte counts but its deficiency in leukocytes impairs cell migration *via* a CCR5-dependent mechanism.

## Introduction

Atherosclerosis is characterized by deposition of cholesterol and chronic inflammation within arterial walls (1, 2). Cholesterol-rich lipoprotein particles accumulate in the sub-endothelial layer of medium- and large-sized arteries, which, in turn, promotes endothelial cell activation and consequent entry of leukocytes into growing atherosclerotic lesions. It has been demonstrated that the development of atherosclerotic lesions correlates directly with an increased number of circulating leukocytes, particularly monocytes, and their enhanced recruitment to inflamed arteries (3, 4). Monocytes in the intima differentiate into macrophages, take up modified lipoprotein particles *via* scavenger receptors and eventually transform into foam cells. These cells have impaired migratory capacity and tend to persist in atherosclerotic plaques, thus promoting disease progression (5–8). Hypercholesterolemia is an important risk factor for atherosclerosis, since it not only drives lipid deposition in lesions but it also induces an expansion of circulating monocytes (6). This is attributable to an increase in the classical, pro-inflammatory Ly6C^high^ monocyte subset and is primarily mediated by increased proliferation of hematopoietic stem cells and their release from the bone marrow. In addition to cholesterol, hematopoiesis is induced by sympathetic activation and production of proinflammatory cytokines that follow acute cardiovascular events such as myocardial infarction (9). While hematopoiesis occurs mainly in the bone marrow under healthy steady-state conditions, atherosclerosis-linked inflammation can stimulate myeloid cell production in other lymphoid organs, particularly in the spleen (10). Monocytes from the bone marrow and spleen are released into the circulation, extravasate and accumulate as macrophages in the plaque. Although macrophages predominate in the lesions, T cells are also abundant and contribute significantly to the initiation and progression of atherosclerosis (11). CD4^+^ T cells are the most frequent T cell subset in the plaque and their disease-promoting activity is especially ascribed to the interferon-γ (IFN-γ)-producing T helper type 1 (Th1) subset (12, 13). In contrast, regulatory T cells (Tregs) are atheroprotective (14), while the role of Th2 cells is more controversial based on observations of both pro- and anti-atherosclerotic effects (11). Evidence thereby strongly supports the involvement of both innate and adaptive immunity in atherogenesis.

We have recently found that melanocortins are implicated in the regulation of macrophage function and cholesterol homeostasis in atherosclerosis (15, 16). Melanocortins are products of post-translational processing of the pro-opiomelanocortin (POMC) precursor protein and include melanocyte-stimulating hormones (α-, β- and γ-MSH) and adrenocorticotropic hormone (ACTH) (17). They interact with five closely related G-protein coupled melanocortin receptor (MC1-R - MC5-R), which regulate important physiological functions including skin pigmentation, steroidogenesis and energy homeostasis (18). MC1-R is the best-characterized regulator of skin and hair coloration but it also plays a significant role in the control of inflammation (19–21). It has a wide expression profile in the immune system and is present virtually in all major leukocyte subpopulations including neutrophils, monocytes, macrophages, dendritic cells, cytotoxic T cells and B lymphocytes (22–25). It is well-established that MC1-R exerts potent anti-inflammatory actions (21), making it an attractive therapeutic target in inflammatory diseases such as atherosclerosis. Indeed, MC1-R activation was found to be protective in experimental models of atherosclerosis (15, 26, 27), but the underlying mechanism is likely to be multifactorial and extends beyond the immunoregulatory function of MC1-R. We recently identified a novel role for MC1-R in macrophage cholesterol transport, which provides protection against atherosclerosis by inhibiting foam cell formation (15). Conversely, phenotyping of recessive yellow Mc1r^e/e^ mice that carry a single base deletion mutation in the Mc1r gene revealed that MC1-R deficiency exacerbates atherosclerosis by disturbing cholesterol and bile acid metabolism and by increasing arterial accumulation of pro-inflammatory Ly6C^high^ monocytes (16). However, the observed phenotype cannot be solely attributed to loss-of-function of MC1-R in leukocytes, since this receptor is distributed also in other tissues relevant to atherosclerosis such as the liver, endothelium and adipose tissue (28–31). Therefore, the exact role of leukocyte MC1-R in the development of atherosclerosis remains to be determined. In this study, we set out to address this and transplanted apolipoprotein E knockout (Apoe^-/-^) mice with MC1-R deficient bone marrow and then analyzed tissue leukocyte profiles and plaque phenotype of this mouse model.

## Materials and Methods

### Mice

C57BL/6 Mc1r^e/e^ recessive yellow mice (Jackson Laboratory, strain# 000060, Bar Harbor, ME, USA) were intercrossed with C57BL/6 Apoe^-/-^ mice (Jackson Laboratory, strain# 002052) to generate double mutant Apoe^-/-^ Mc1r^e/e^ mice. Mice were housed in groups of 3-5 littermates on a 12 h light/dark cycle. The experiments were approved by the local ethics committee (Animal Experiment Board in Finland, License Numbers: ESAVI/6280/04.10.07/2016 and ESAVI/1260/2020) and conducted in accordance with the institutional and national guidelines for the care and use of laboratory animals.

### Bone Marrow Transplantation

Bone marrow (BM) cells were isolated from the femurs and tibiae of female Apoe^-/-^ Mc1r^e/e^ mice or age-matched Apoe^-/-^ mice. Eight- to ten-week-old recipient Apoe^-/-^ male mice were lethally irradiated with two doses of 5 Gy 3 hours apart in a Faxitron MultiRad 225 X-ray irradiator (32). Three days later, recipient mice were reconstituted intravenously with 1x10^7^ BM cells from either Apoe^-/-^ or Apoe^-/-^ Mc1r^e/e^ mice. Mice received acidified and autoclaved water from 1 week prior to the BM transplantation until 4 weeks after. Mice were allowed to recover for 6 weeks and were thereafter either maintained on a normal chow diet or fed a Western-type diet (D12079B, Research Diets Inc, NJ, USA) for 10 weeks. At the end of the experiment, mice were euthanized *via* CO2 asphyxiation and whole blood was obtained *via* cardiac puncture. Aorta, spleen, liver and bone marrow (femur) were collected for further analyses.

To determine the efficiency of BM reconstitution, genomic DNA was extracted (QIAamp DNA Blood Mini Kit, Qiagen, USA) from peripheral blood of recipient mice at the end of the experiment. Samples were quantified by real-time PCR (Applied Biosystems 7300 Real-Time PCR system) for the expression of the Y chromosome-specific gene Zfy1 and a reference gene (Bcl2) (33). The engraftment of female donor cells in the recipient males was calculated using a standard curve generated from samples with known percentages of male and female DNA. At 16 weeks after BM transplantation, donor cell engraftment level in the peripheral blood ranged from 95% to 99%. The level of chimerism was 97.2 ± 0.3% in Apoe^-/-^ and 97.6 ± 0.4% in Apoe^-/-^ Mc1r^e/e^ BM transplanted mice (P=0.48).

### Flow Cytometry

Total leukocytes and leukocyte subsets in the aorta, spleen, bone marrow (femur) and whole blood were quantified by flow cytometry as previously described (16). Aortic samples were digested with an enzymatic cocktail (Collagenase I, 450 U/ml; Collagenase XI, 250 U/ml; Hyaluronidase, 120 U/ml; DNase I, 120 U/ml; Sigma Aldrich) for 60 min at 37°C and then filtered through a 50-µm cell strainer (BD Biosciences). Single cell suspensions were stained for 30 min at 4°C with fluorochrome-conjugated antibodies against CD45.2 (clone 30-F11, BD Biosciences), CD11b (clone M1/70, BioLegend), CD115 (clone AFS98, BioLegend), Ly6C (clone AL-21, BD Biosciences) and Ly6G (clone 1A8, BD Biosciences). To quantify lymphocyte subsets, cells were stained with CD45.2 (clone 30-F11, BD Biosciences), CD11b (clone M1/70, BioLegend), CD19 (clone 6D5, Biolegend) CD4 (GK1.5, Biolegend), CD8a (clone 53-6.7, Biolegend), NK1.1 (clone PK136, Biolegend) and TCR-β (clone H57-597). To stain intracellular antigens, splenocytes were harvested and incubated in RPMI 1640 medium supplemented with cell stimulation and protein transport inhibitor cocktail (eBioscience, catalogue number: 00-4975-03), 10% fetal bovine serum and 100 U/100 μg/ml penicillin-streptomycin (Gibco Life Technologies, NY, USA) for 18 hours. Thereafter, cells were washed with PBS, stained for surface antigens (CD45, CD11b, TCR-β and CD4), fixed and permeabilized (eBioscience, catalogue number: 00-5523-00) and then stained with antibodies against IFN-γ (clone XMG1.2, Biolegend) and FoxP3 (clone JJK-16s, eBioscience). To analyze hematopoietic stem cells, bone marrow suspensions were stained with antibodies against lineage markers (cocktail of Ter119, B220, CD11b, CD3, and GR1), c-Kit (clone 2B8), Sca-1 (clone D7), CD48 (clone HM48-1) and CD150 (clone TC15-12F12.2, all from BioLegend). Data were acquired on an LSR Fortessa (BD Biosciences) and the results were analyzed with FlowJo software (FlowJo, LLC, Ashland, USA).

### *In Vivo* Homing Assay

Splenocytes or bone marrow cells from male Apoe^-/-^ and Apoe^-/-^ Mc1r^e/e^ mice were harvested and red blood cells were lysed (BD Pharm Lyse™, BD Biosciences) and thereafter, the samples were washed and filtered through a 70-μm cell strainer. Splenocytes and bone marrow cells were stained with eFluor™ 670 (eBioscience, catalogue number: 65-0840-85) or carboxyfluorescein succinimidyl ester (CFSE, Invitrogen™, catalogue number: C34554) at 37°C for 15 min. Cells were washed with RPMI 1640 medium containing 10% FBS, resuspended in PBS and mixed at 1:1 ratio. Ten million splenocytes or bone marrow cells were injected *via* tail vein into each recipient male Apoe^-/-^ mouse. Twenty-four hours after the injection, mice were sacrificed and blood, spleen and para-aortic lymph nodes were harvested for staining with antibodies against CD45, CD11b, TCR-β, CD4 and CD8 or CD45, CD11b, CD115, Ly6C and Ly6G. Samples were analyzed by flow cytometry (LSR Fortessa, BD Biosciences) and the results are expressed as percentage of injected CD45^+^ cells.

### CD4^+^ T Cell Isolation

Spleens from Apoe^-/-^ and Apoe^-/-^ Mc1r^e/e^ mice were harvested and processed into single cell suspensions as previously described (15). Splenic CD4^+^ T cells were then isolated by positive selection (Invitrogen, catalogue number: 8802-6841-74) and the resulting cell fraction was subjected to total RNA or protein extraction.

### Chemotaxis Assay

Splenocytes from Apoe^-/-^ and Apoe^-/-^ Mc1r^e/e^ mice were harvested and subjected to chemotaxis assay using 6.5 mm Transwell® polycarbonate membrane cell culture inserts (5.0-μm pore size, Corning, NY, USA). In brief, splenocytes were harvested as described above and suspended in RPMI1640 medium supplemented with 0.5% fatty acid-free BSA. Chemokine-containing (murine recombinant CCL3, CCL4 or CCL5, 100 ng/mL, PeproTech, London, UK) migration medium (RPMI-1640 + 0.5% fatty acid-free BSA) was placed in the bottom of the well and thereafter, an aliquot (100 μL, 1x10^6^ cells) of cell suspension was seeded in the top chamber. After 3-hour incubation at 37°C in a 5% CO_2_ atmosphere, the top chamber was removed and migrated cells were stained (CD45, TCR-β, CD4, CD8, CD115, Ly6C and Ly6G) and counted by flow cytometry. The number of migrated cells is expressed as percentage of CD45^+^ cells that were seeded in the top chamber.

### CCR5 Internalization and Recycling Assay

Splenocytes were harvested from Apoe^-/-^ and Apoe^-/-^ Mc1r^e/e^ mice and suspended in RPMI1640 medium containing 0.5% fatty acid-free BSA at a density of 1 x 10^6^ cells/mL. After 60 min incubation at 37°C, cells were left untreated or stimulated with recombinant murine CCL5 (400 ng/mL, 60 min, PeproTech) to evoke internalization of CCR5. To evaluate recycling of CCR5, CCL5-stimulated cells were washed three times with RPMI1640 medium, resuspended in RPMI1640 medium containing 0.5% fatty acid-free BSA and incubated for 60 min at 37°C. Cells were harvested, stained (CD45, TCR-β, CD4, CD8 and CCR5, clone HM-CCR5, Biolegend) and analyzed for the expression of cell surface CCR5 by flow cytometry.

### RNA Isolation, cDNA Synthesis and Quantitative RT-PCR

Spleen, bone marrow and CD4^+^ T cell samples were isolated and homogenized in QIAzol Lysis Reagent using the Qiagen TissueLyser LT Bead Mill (QIAGEN, Venlo, Netherlands). Total RNA was extracted (Direct-zol RNA Miniprep, Zymo Research, CA, USA) and reverse-transcribed to cDNA with PrimeScript RT reagent kit (Takara Clontech) according to the manufacturer’s instructions. Quantitative real-time polymerase chain reaction (RT-PCR) was performed with SYBR Green protocols (Kapa Biosystems, MA, USA) and a real-time PCR detection system (Applied Biosystems 7300 Real-Time PCR system). Samples were run in duplicate. Target gene expression was normalized to the geometric mean of two housekeeping genes (ribosomal protein S29 and β-actin) using the delta-Ct method and results are presented as relative transcript levels (2^-ΔΔCt^). Primer sequences are presented in **Supplementary Table 1**.

### Histology and Immunohistochemistry

Aortic roots were fixed in 10% formalin overnight followed by embedding in paraffin and cutting into in 4 μm-thick serial sections. Sections were stained with hematoxylin and eosin (H&E) and Masson’s trichrome to measure atherosclerotic plaque area, the size of necrotic core and plaque collagen content at the level of the aortic sinus. For immunohistochemistry, sections were incubated in 10 mM sodium citrate buffer (pH 6) for 20 min in a pressure cooker for antigen retrieval. Thereafter, sections were blocked in 1% H_2_O_2_ for 20 min and then in 10% normal horse serum. Samples were incubated overnight with primary antibodies against Mac-2 (1:300, Abcam) or alpha smooth muscle actin (α-SMA, 1:200, Sigma-Aldrich, St. Louis, MO, USA) followed by horseradish peroxidase-conjugated secondary antibody incubation and detection with diaminobenzidine (ABC kit, Vector Labs, Burlingame, USA) to estimate macrophage- and smooth muscle cell-positive plaque areas, respectively. For immunofluorescence, frozen 6 μm-thick spleen sections were stained with antibodies against CD4 (1:50, clone RM4-5, Biotechne) and MC1-R (1:50, Elabscience) followed by detection with fluorochrome-conjugated secondary antibodies (anti-rabbit Alexa Fluor 647 or anti-rat Alexa Fluor 488 Jackson ImmunoResearch, West Grove, USA). For the multicolor immunofluorescence imaging, negative and single-stain controls were included and are presented in **Supplementary Figure 1**. Sections were counterstained with hematoxylin (CarlRoth) or DAPI (Fluoroshield mounting medium, Abcam), cover-slipped and then scanned with Pannoramic 250 or Pannoramic Midi digital slide scanner (3DHISTECH Kft, Budapest, Hungary). Image analyses was perform using ImageJ software (NIH, Bethesda, MD, USA).

### Plasma Cholesterol Assay and Cytokine Analysis

Plasma lipids and lipoproteins were obtained from EDTA-anticoagulated whole blood and measured with commercial enzymatic colorimetric assays (CHOD-PAP and GPO-PAP, mti Diagnostics, Idstein, Germany) according to the manufacturer’s protocols. Plasma total cholesterol concentration was determined. Plasma pro-inflammatory cytokines and chemokines as well as plasma antibodies were quantified with ProcartaPlex™ Multiplex Immunoassays (High Sensitivity 5-Plex Mouse Panel, catalogue number: EPXS050-22199-901, eBioscience & Mouse Antibody Isotyping Panel, catalogue number: EPX070-20815-901, Thermo Fisher Scientific) (34).

### Western Blotting

CD4^+^ sorted cell samples were lysed in RIPA containing a protease inhibitor cocktail (Complete Mini, Roche). Aliquots of total protein were separated by SDS-PAGE and transferred to a nitrocellulose membrane. Blots were probed with antibodies against CCR5 (Novus Biologicals, Bio-techne Ltd, UK) and MC1-R (Alomone Labs, Jerusalem, Israel). Horseradish peroxidase-conjugated anti-IgG (Cell Signaling Tech, Frankfurt, DE) secondary anti-body detection was applied and membranes were developed using a chemiluminescence system (ECL detection reagent: Pierce ECL Western (Thermo Scientific, USA).

### Statistics

Statistical analyses were performed with GraphPad Prism 8 software (La Jolla, CA, USA). Statistical significance between the experimental groups was determined by unpaired Student’ t-test or two-way ANOVA followed by Bonferroni *post hoc* tests. The D’Agostino and Pearson omnibus normality test method was employed to test the normality of the data. Possible outliers in the data sets were identified using the regression and outlier removal (ROUT) method at Q-level of 1%. Data are expressed as mean ± standard error of the mean (SEM). Results were considered significant for P<0.05.

## Results

### Hematopoietic MC1-R Deficiency Induced Leukocytosis in Apoe^-/-^ Mice

**Figure 1:**
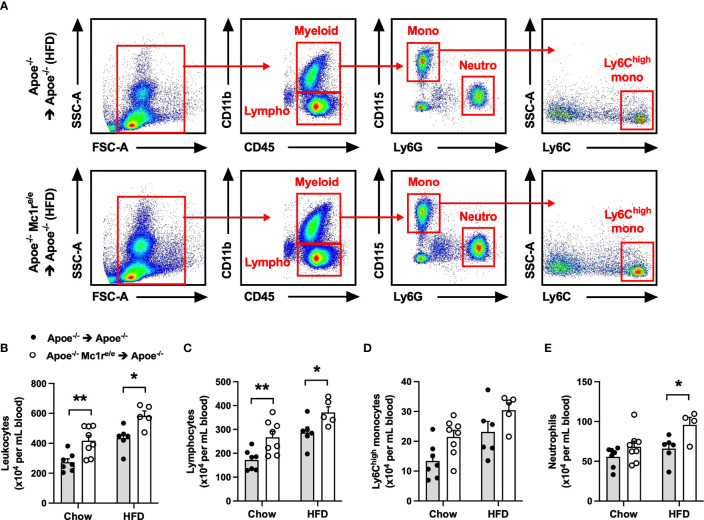
Hematopoietic MC1-R deficiency induced leukocytosis in Apoe^-/-^ mice. **(A)** Representative dot plots for the gating of total leukocytes (CD45^+^), lymphocytes (CD45^+^, CD11b^-^), neutrophils (CD45^+^, CD11b^+^, CD115^-^ Ly6G^+^) and Ly6C^high^ monocytes (CD45^+^, CD11b^+^, CD115^+^, Ly6C^high^) in the peripheral blood of Apoe^-/-^ and Apoe^-/-^ Mc1r^e/e^ BM transplanted mice. **(B–E)** Quantification of total leukocyte, lymphocyte, Ly6C^high^ monocyte and neutrophil counts in the blood of Apoe^-/-^ and Apoe^-/-^ Mc1r^e/e^ BM transplanted mice fed a chow or high-fat diet (HFD) for 10 weeks. Data are mean ± SEM, *P < 0.05, **P < 0.01 versus Apoe^-/-^ mice. Each dot represents individual mouse. SSC-A indicates side scatter area; FSC-A, forward scatter area; Apoe, apolipoprotein E.

To determine the contribution of leukocyte MC1-R to atherosclerosis, we transplanted Apoe^-/-^ or Apoe^-/-^ Mc1r^e/e^ BM into lethally-irradiated Apoe^-/-^ recipient mice. After transplantation and recovery period, mice were maintained on chow diet or fed a cholesterol-rich high fat diet (HFD) for 10 weeks to enhance hypercholesterolemia and atherosclerosis. Hematopoietic MC1-R deficiency did not affect body weight or plasma cholesterol concentration in either of the diet groups (**Supplementary Table 2**). Given the importance of leukocytes and inflammation in the development of atherosclerosis, we did a thorough immunophenotyping for the blood, spleen and BM by flow cytometry. Of note, total leukocyte count was significantly increased in the blood of Apoe^-/-^ Mc1r^e/e^ BM transplanted mice (**Figures 1A, B**). This effect was observed in both diet groups and was largely attributable to increased lymphocyte count (**Figure 1C**). The numbers of circulating Ly6C^high^ monocytes and neutrophils were also elevated (P=0.008 and 0.006, respectively, for genotype effect by 2-way ANOVA) in Apoe^-/-^ Mc1r^e/e^ chimeras (**Figures 1D, E**). Further phenotyping of lymphocyte subsets revealed increased B and CD4^+^ T cell counts in the blood of Apoe^-/-^ Mc1r^e/e^ chimeric mice (**Supplementary Figure 2**), while the numbers of NK cells, NK T cells and CD8^+^ T cells were comparable between the experimental groups. In agreement with increased B cell count, we observed an elevation in plasma antibody concentrations particularly in HFD-fed Apoe^-/-^ Mc1r^e/e^ chimeric mice (**Supplementary Figure 2**). This effect was evident across different antibody subclasses including IgG1, IgG2a, IgG2b, IgA and IgM.

**Figure 2:**
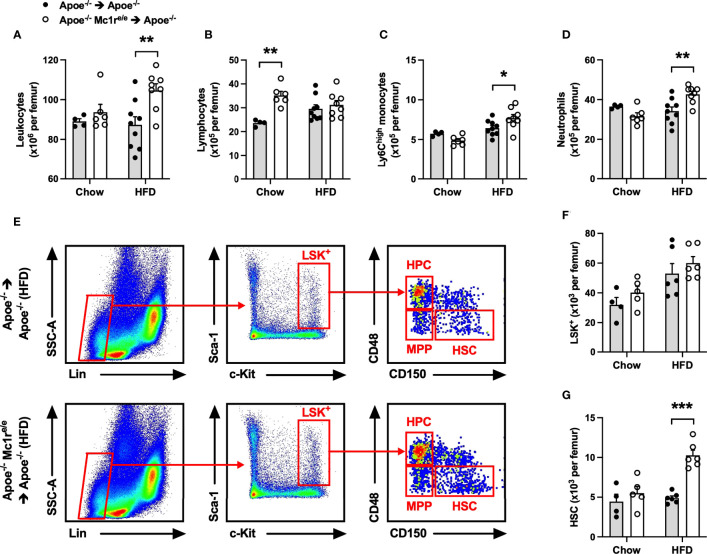
Hematopoietic MC1-R deficiency increased leukocyte and hematopoietic stem cell counts in the bone marrow of Apoe^-/-^ recipient mice. **(A–D)** Quantification of total leukocytes (CD45^+^), lymphocytes (CD45^+^, CD11b^-^), neutrophils (CD45^+^, CD11b^+^, Ly6G^+^) and Ly6C^high^ monocytes (CD45^+^, CD11b^+^, CD115^+^and Ly6C^high^) in the bone marrow of Apoe^-/-^ and Apoe^-/-^ Mc1r^e/e^ chimeric mice. **(E)** Representative dot plots for the gating of LSK^+^ (Lin^-^, Sca-1^+^, c-Kit^+^), MPP (CD48^+^, CD150^-^), HPC (CD48^-^, CD150^-^), HSC (CD150^+^, CD48^-^) cells in the bone marrow of Apoe-/- recipient mice. **(F, G)** Quantification of LSK^+^ and HCS (Lin1^-^, Sca-1^+^, Kit^+^; and CD150^+^, CD48^-^) cells in the bone marrow. Data are mean ± SEM, *P ≤ 0.05, **P < 0.01, ***P < 0.001 versus Apoe^-/-^ mice. Each dot represents individual mouse. Lin^-^ indicates lineage-negative; Sca-1, stem-cell antigen-1; c-Kit, proto-oncogene receptor tyrosin kinase; MPP, multi-potent progenitor; HSC, hematopoietic stem cell; HPC, hematopoietic progenitor cell.

To investigate whether induction of hematopoiesis accounts for the observed leukocytosis in Apoe^-/-^ Mc1r^e/e^ chimeric mice, total leukocytes and different leukocyte subsets as well as hematopoietic stem and progenitor cells were quantified in the bone marrow. We found that HFD feeding had triggered an expansion of total leukocyte, Ly6C^high^ monocyte and neutrophil populations in the bone marrow of Apoe^-/-^ Mc1r^e/e^ BM transplanted mice, while the numbers of these cells were unchanged in chow-fed mice (**Figures 2A, C, D** and **Supplementary Figure 3**). Chimeric Apoe^-/-^ Mc1r^e/e^ mice did not show any change in the frequency of Lin^−^ Sca1^+^ cKit^+^ cells (LSK^+^) that are considered to represent the hematopoietic stem/progenitor cell (HSPC) population (**Figures 2E, F**). However, the number of true HSPCs that are capable of repopulating the hematopoietic system and called long-term hematopoietic stem cells (HSCs; defined as CD150^+^ CD48^-^LSK^+^) was markedly increased in the bone marrow of Apoe^-/-^Mc1r^e/e^ chimeric mice after feeding HFD (**Figure 2G**). This was mechanistically associated with down-regulation of ATP-binding cassette transporter ABCA1 (P=0.08), vascular cell adhesion molecule 1 (VCAM-1) and C-X-C motif chemokine ligand 12 (CXCL12) (**Supplementary Figure 4**), which regulate HSC proliferation and release of progenitor cells into circulation (35–37). On the other hand, chow-fed Apoe^-/-^ Mc1r^e/e^ chimeric mice displayed more multipotent progenitors (MPP; defined as CD150^-^ CD48^-/low^ LSK^+^) (**Supplementary Figure 4**) that are derived from short-term HSCs and can support hematopoiesis transiently (38). This, in turn, might explain the increase in lymphocyte count that was observed only in chow-fed Apoe^-/-^ Mc1r^e/e^ chimeric mice (**Figure 2B**) and stemmed from higher CD4^+^ and CD8^+^ T cell counts (**Supplementary Figure 4**).

### Transplanted Apoe^-/-^ Mc1r^e/e^ BM Caused an Expansion of Splenic CD4^+^ T Cells

**Figure 3:**
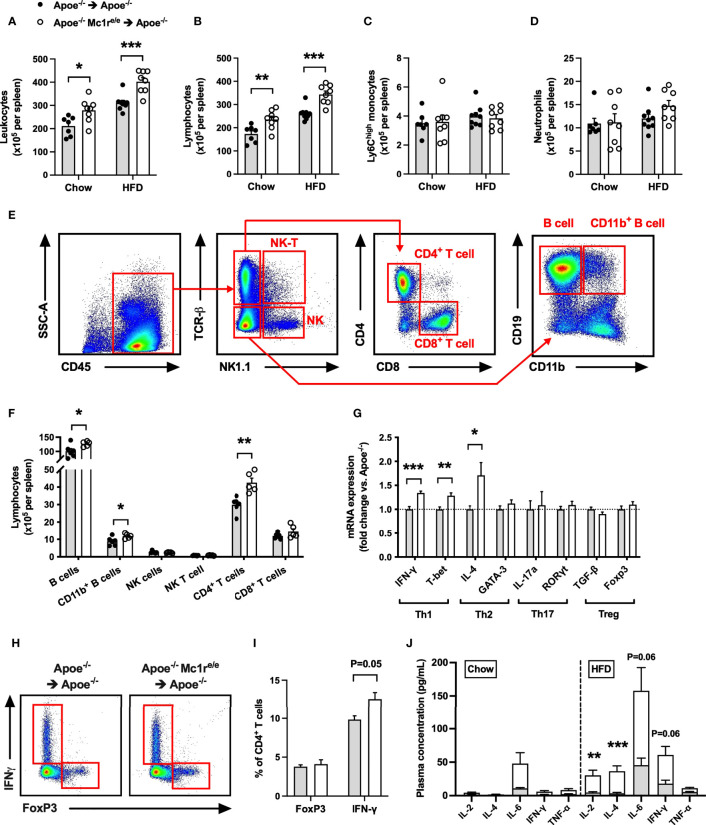
Increased leukocyte and lymphocyte counts in the spleen of Apoe^-/-^ mice reconstituted with MC1-R deficient BM. **(A–D)** Quantification of total leukocytes (CD45^+^), lymphocytes (CD45^+^, CD11b^-^), Ly6C^high^ monocytes (CD45^+^, CD11b^+^, CD115^+^and Ly6C^high^) and neutrophils (CD45^+^, CD11b^+^, Ly6G^+^) in the spleen of Apoe^-/-^ recipient mice. **(E)** Representative dot plots for the gating of NK T cells (CD45^+^, TCRβ^+^, NK1.1^+^), NK cells (CD45^+^, TCRβ^-^, NK1.1^+^), CD4^+^ T cells (CD45^+^, TCRβ^+^, CD4^+^), CD8^+^ T cells (CD45^+^, TCRβ^+^, CD8^+^), B cells (CD45^+^, TCRβ^-^, CD19^+^, CD11b^-^) and CD11b^+^ B cells (CD45^+^, TCRβ^-^, CD19^+^, CD11b^+^) in the spleen of Apoe^-/-^ BM transplanted mouse. **(F)** Quantification of splenic lymphocyte subsets in HFD-fed recipient mice. **(G)** Quantitative real-time PCR (qPCR) analysis of effector CD4^+^ T cell markers in the spleen of Apoe^-/-^ and Apoe^-/-^ Mc1r^e/e^ BM transplanted mice. n=8-9 mice per genotype. **(H, I)** Representative flow cytometry results for the gating and quantification of IFN-γ^+^ and Foxp3^+^ CD4^+^ T cells (CD45^+^, TCRβ^+^, CD4^+^) in the spleen of Apoe^-/-^ and Apoe^-/-^ Mc1r^e/e^ BM transplanted mice. n=4 mice per genotype. **(J)** Pro-inflammatory cytokine concentrations in the plasma of chow- and HFD-fed Apoe-/^-^ mice. *P < 0.05, **P < 0.01, ***P < 0.001 versus control Apoe^-/-^ mice. Data are mean ± SEM.

Based on the heightened leukocyte count, particularly lymphocyte count, observed in the blood of Apoe^-/-^ Mc1r^e/e^ chimeric mice, we next sought to quantify leukocytes and their subsets in the spleen, which constitutes an important source of inflammatory leukocytes infiltrating atherosclerotic plaques. Consistently, flow cytometry analysis revealed an increased leukocyte count in the spleen of Apoe^-/-^ Mc1r^e/e^ chimeric mice (**Figure 3A** and **Supplementary Figure 5**). This effect was also reflected as higher spleen weight in HFD-fed Apoe^-/-^ Mc1r^e/e^ chimeric mice (**Supplementary Table 2**). The increased leukocyte count was attributable to augmented total lymphocyte count in Apoe^-/-^ Mc1r^e/e^ BM engrafted mice (**Figure 3B**), while Ly6C^high^ monocyte and neutrophil counts were comparable between the genotypes (**Figures 3C, D**). Identification and enumeration of different lymphocyte subsets revealed that MC1-R deficient mice had significantly higher B cell and CD4^+^ T cell counts in the spleen (**Figures 3E, F** and **Supplementary Figure 6**). It is worth noting that CD4^+^ T cell count was consistently increased in the blood, bone marrow and spleen of Apoe^-/-^mice receiving MC1-R deficient BM. Since this cell population comprises different effector cells, including Th1, Th2, Th17 and Treg cells, that can have opposing effects on atherosclerosis, we did a qPCR analysis and screened the spleen samples for the signature markers of different CD4^+^ T cell subtypes. Apoe^-/-^ Mc1r^e/e^ chimeric mice displayed upregulation of the Th1 cytokine interferon gamma (IFN-γ) and the transcription factor T-bet that drive the differentiation of CD4^+^ T cells into Th1 effector cells (**Figure 3G**). The expression of the Th2 cytokine interleukin 4 (IL-4) was also upregulated but it was not accompanied by transcriptional changes in GATA3 that polarize cells to a Th2 phenotype. None of the Th17 or Treg signature genes were affected in Apoe^-/-^ Mc1r^e/e^ chimeric mice. These phenotypic characteristics were further confirmed by flow cytometry analysis that indicated an increase in the proportion of IFN-γ-expressing CD4^+^ T cells (**Figures 3H, I**). Additionally, plasma concentrations of the T cell growth factor interleukin 2 (IL-2) and IL-4 were significantly increased in Apoe^-/-^ Mc1r^e/e^ chimeric mice on HFD (**Figure 3J**). IL-6 and IFN-γ concentrations were also moderately elevated (P=0.06) in the plasma (**Figure 3J**) of these mice. Taken together, bone marrow transplantation of Apoe^-/-^ Mc1r^e/e^ BM into Apoe^-/-^ mice expanded CD4^+^ T cell population and the Th1 effector cells appeared to be the primary CD4^+^ T cell subtype responsible for this expansion.

### Enhanced Leukocytosis in Apoe^-/-^ Mc1r^e/e^ Chimeric Mice Was Not Associated With Accelerated Atherosclerosis

**Figure 4:**
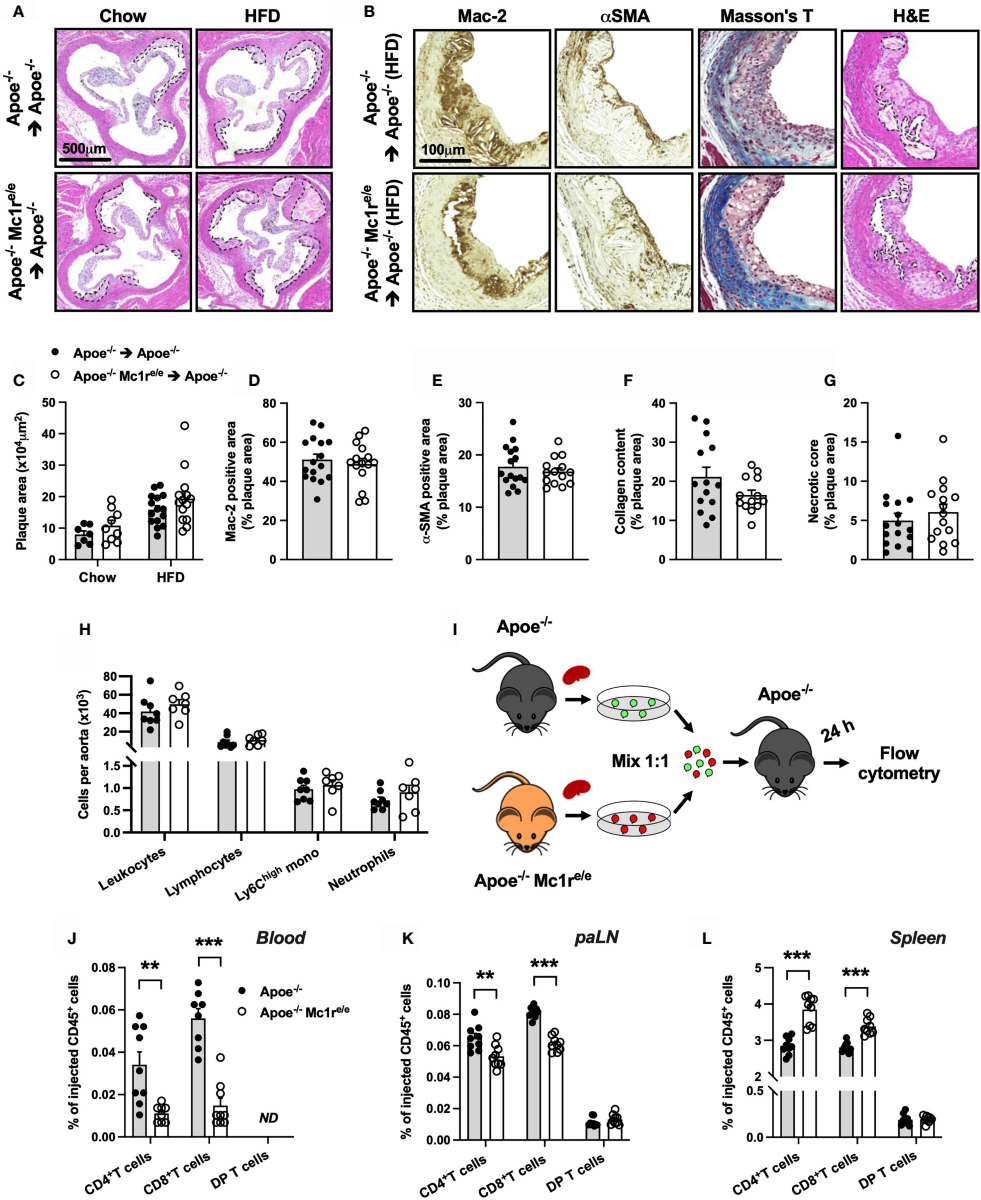
Hematopoietic MC1-R deficiency does not affect plaque phenotype but significantly alters homing behavior of T cells. **(A)** Representative images of hematoxylin and eosin (H&E) staining of the aortic sinus of Apoe^-/-^ and Apoe^-/-^ Mc1r^e/e^ BM engrafted mice on chow or HFD for 10 weeks. **(B)** Representative images of Mac-2 (galectin-3), α-SMA (α-smooth muscle actin), Masson trichrome staining and necrotic core areas in the aortic sinus. Necrotic core areas are indicated with dashed lines in H&E-stained images. **(C)** Quantification of plaque area in aortic sinuses. **(D–G)** Quantification of Mac-2- and α-SMA – positive areas, plaque collagen content and acellular necrotic core areas as percentage of total plaque area. **(H)** Quantification of total leukocytes (CD45^+^), lymphocytes (CD45^+^, CD11b^-^), Ly6C^high^ monocytes (CD45^+^, CD11b^+^, CD115^+^, Ly6C^high^) and neutrophils (CD45^+^, CD11b^+^, Ly6G^+^) by flow cytometry in the aorta of Apoe^-/-^ recipient mice. **(I)** Experimental design for analyzing homing of different lymphocyte subsets to the para-aortic lymph nodes (paLN) and spleen. Cells were isolated from the spleen of Apoe^-/-^ and Apoe^-/-^ Mc1r^e/e^ mice and injected into recipient Apoe^-/-^ mice. **(J–L)** Quantification of CD4^+^ T cells (CD45^+^, TCRβ^+^, CD4^+^), CD8^+^ T cells (CD45^+^, TCRβ^+^, CD8^+^) and double positive (DP) T cells (CD45^+^, TCRβ^+^, CD4^+^, CD8^+^) by flow cytometry in the blood, paLNs and spleen as percentage of injected CD45^+^ cells. Data are mean ± SEM, **P < 0.01, ***P < 0.001 versus control Apoe^-/-^ mice. Each dot represents individual mouse.

We next aimed to address whether enhanced leukocytosis in Apoe^-/-^ Mc1r^e/e^ chimeric mice affects atherosclerosis, and analyzed the development of atherosclerotic lesions at the aortic root. Unexpectedly, Apoe^-/-^ Mc1r^e/e^ BM transplantation did not change plaque size in chow- or HFD-fed mice (P=0.10 for genotype effect by 2-way ANOVA) (**Figures 4A, C**). Further characterization of atherosclerotic lesions in HFD-fed mice did not reveal any significant changes in terms of plaque macrophage (**Figures 4B, D**) or smooth muscle cell (**Figures 4B, E**) content as judged by the expression levels of Mac-2 and α-smooth muscle actin (α-SMA). In addition, plaque collagen content and necrotic core area were comparable between the genotypes (**Figures 4B, F, G**). Lastly, flow cytometric analysis of aortic lysates showed that Apoe^-/-^ Mc1r^e/e^ BM transplantation did not result in enhanced accumulation of total leukocytes, lymphocytes, Ly6C^high^ monocytes or neutrophils in the aorta (**Figure 4H**). These findings contradict the observed phenotype in the blood, BM and spleen of Apoe^-/-^ Mc1r^e/e^ chimeric mice, characterized by markedly increased leukocyte counts.

### Homing of CD4^+^ and CD8^+^ T Cells to Para-Aortic Lymph Nodes Is Compromised in the Absence of MC1-R

The apparent mismatch between the expected and observed plaque phenotype raised a question whether MC1-R deficient leukocytes have an altered migratory behavior. We therefore performed an *in vivo* homing assay with CD45^+^ cells isolated from the spleen of male Apoe^-/-^ and Apoe^-/-^ Mc1r^e/e^ mice (**Figure 4I**). Cells were fluorescently labeled and injected into recipient male Apoe^-/-^ mice and quantified by flow cytometry in the blood, para-aortic lymph nodes (paLNs) and spleen 24 hours after the injection. In the blood and paLNs, we observed a significant reduction in the number of CD4^+^ and CD8^+^ T cells that were isolated from Apoe^-/-^ Mc1r^e/e^ mice compared to control Apoe^-/-^ cells (**Figures 4J, K** and **Supplementary Figure 7**). In contrast, no difference was noted in double positive CD4^+^ CD8^+^ T cells (DP T cells) that were used as internal control to confirm that the same effect is not universally appearing in all cell types (**Figures 4J–L**). Instead of being retained in the blood and homing into paLNs, CD4^+^ and CD8^+^ T cells from Apoe^-/-^ Mc1r^e/e^ mice were trafficking more into the spleen (**Figure 4L**). We also performed a similar homing assay using myeloid cell-enriched BM from Apoe^-/-^ and Apoe^-/-^ Mc1r^e/e^ mice to track the migration of monocytes and neutrophils (**Supplementary Figure 8**). Mimicking the behavior of CD4^+^ and CD8^+^ T cells, classical Ly6C^high^ and patrolling Ly6C^low^ monocytes from Apoe^-/-^ Mc1r^e/e^ mice migrated more readily into the spleen in comparison with the corresponding cell types from Apoe^-/-^ mice (**Supplementary Figure 8**). Ly6C^high^ and Ly6C^low^ monocytes from Apoe^-/-^ Mc1r^e/e^ mice were also retained more in the circulation (**Supplementary Figure 8**), while in the paLNs, they were detected in equal amounts as control Apoe^-/-^ cells (**Supplementary Figure 8**). On the other hand, neutrophils did not show any genotype difference in their migratory behavior (**Supplementary Figures 8**). Collectively, MC1-R deficient CD4^+^ and CD8^+^ T cells as well as monocytes preferentially home to the spleen and are thereby less likely to migrate to the aorta.

### MC1-R Is Expressed on CD4^+^ T Cells and Regulates mRNA and Protein Level of the Chemokine Receptor CCR5

**Figure 5:**
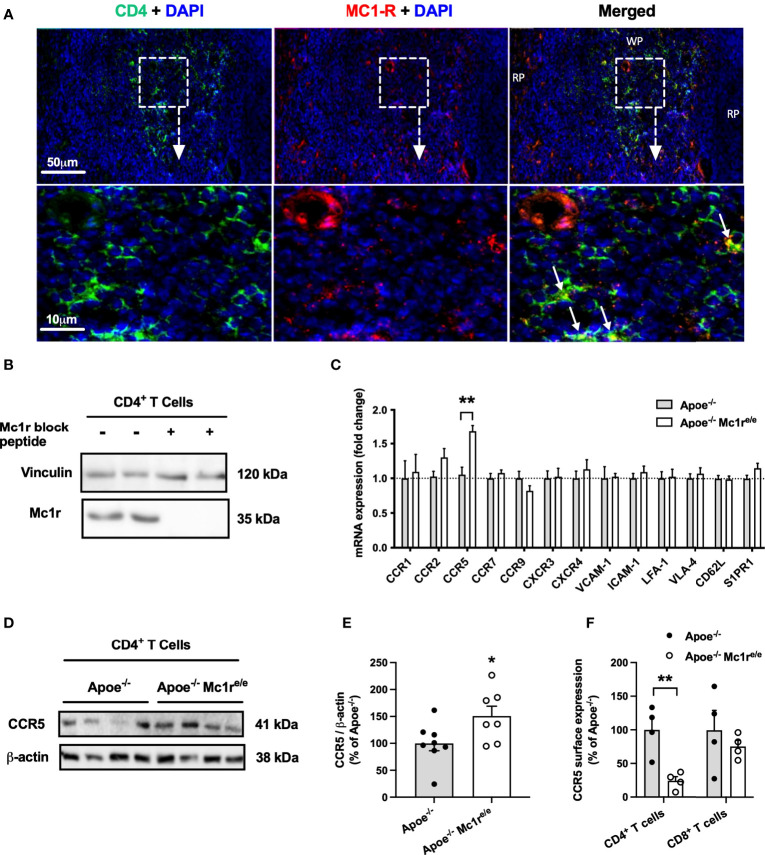
| MC1-R is expressed on splenic CD4^+^ T cells and its deficiency selectively modulates CCR5 expression. **(A)** Immunofluorescence staining of MC1-R and CD4 in the mouse spleen. White arrows indicate co-localization of MC1-R and CD4. RP indicates red pulp; WP, white pulp. **(B)** Western blot analysis of MC1-R protein expression in isolated CD4^+^ T cell samples from the spleen. The expression of vinculin is shown as loading control. **(C)** Quantitative real-time PCR (qPCR) analysis of chemokine receptor and adhesion molecule expression in isolated CD4^+^ T cells from Apoe^-/-^ or Apoe^-/-^ Mc1r^e/e^ mice. Lanes on the right were incubated in anti-Mc1r antibody solution that was premixed with a molar excess of a blocking Mc1r peptide **(D, E)** Representative Western blots and quantification of CCR5 and β-actin (loading control) in isolated CD4^+^ T cells lysates from Apoe^-/-^ or Apoe^-/-^ Mc1r^e/e^ mice. **(F)** Quantification of CCR5 surface expression by flow cytometry in CD4^+^ and CD8^+^ T cells from the spleen of Apoe^-/-^ or Apoe^-/-^ Mc1r^e/e^ mice. Data are mean ± SEM, *P < 0.05, **P < 0.01 versus control Apoe^-/-^ mice. Each dot represents individual mouse.

Having noted significantly increased CD4^+^ T cell counts and altered homing behavior of these cells in the Apoe^-/-^ Mc1r^e/e^ genotype, it raised a question of whether these cells express MC1-R. It has been previously reported that MC1-R is expressed in mouse and human CD8^+^ T cells but not in CD4^+^ T cells (25), while another study found high MC1-R mRNA levels particularly in human CD4^+^ T cells (39). It was therefore crucial to establish the possible presence of MC1-R in CD4^+^ T cells. Indeed, double immunofluorescence staining revealed localization of MC1-R in CD4^+^ cells in the murine spleen (**Figure 5A**). Supporting this finding, Western blot analysis using sorted CD4^+^ T cells from the spleen demonstrated a clear expression of MC1-R protein in these cells. (**Figure 5B**). The specificity of the signal was validated by preadsoprtion with a blocking peptide (**Figure 5B**). Furthermore, MC1-R mRNA level was significantly higher in sorted CD4^+^ T cell samples compared to whole spleen or liver (**Supplementary Figure 9**). To find a potential explanation for the different homing behavior, we sorted CD4^+^ T cells from the spleen of Apoe^-/-^ and Apoe^-/-^ Mc1r^e/e^ mice and analyzed the expression of various chemokine receptors and adhesion molecules that are known to regulate lymphocyte migration. Among all screened genes by qPCR, the expression of CCR5 stood out by showing significant upregulation in Apoe^-/-^ Mc1r^e/e^ CD4^+^ T cells (**Figure 5C**). Likewise, protein expression of CCR5 was increased in Mc1r deficient CD4^+^ T cells (**Figure 5D, E**) Given that CCR5 guides T cell recruitment and infiltration into sites of tissue inflammation such as atherosclerotic plaques (40, 41), increased CCR5 mRNA and protein expression in MC1-R deficient CD4^+^ T cells was unexpected and contradicts the finding of preferential homing of these cells into the spleen. To investigate this issue further, we analyzed CCR5 expression at the cell surface of splenic T cells by flow cytometry. Interestingly, CCR5 surface expression as well as percentage of CCR5^+^ cells was significantly reduced in Apoe^-/-^ Mc1r^e/e^ CD4^+^ T cells (**Figure 5F** and **Supplementary Figure 9**). These changes were not evident in CD8^+^ T cells from Apoe^-/-^ Mc1r^e/e^ mice (**Figure 5F** and **Supplementary Figure 9**).

### Dysfunctional MC1-R Impair Recycling of CCR5 in CD4^+^ T Cells

**Figure 6:**
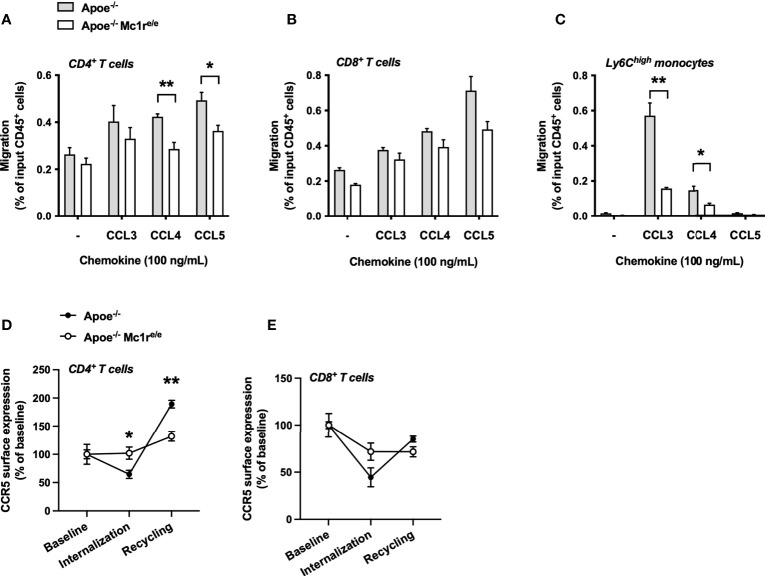
MC1-R deficient CD4^+^ T cells show impaired CCR5-dependent migration and recycling of the CCR5 receptor. **(A–C)** Transwell migration assay using splenocytes from Apoe^-/-^ and Apoe^-/-^ Mc1r^e/e^ mice. Cell were allowed to migrate for 3 hours towards the indicated chemokines and migrated CD4^+^ T cells, CD8^+^ T cells and Ly6C^high^ monocytes were quantified by flow cytometry as percentage of input CD45^+^ cells. n=4 mice per genotype **(D, E)** Quantification of CCR5 surface expression on CD4^+^ cells and CD8^+^ T cells that were isolated from Apoe^-/-^ or Apoe^-/-^ Mc1r^e/e^ mice. CCR5 surface expression was quantified during baseline, after CCL5-induced internalization and after withdrawal of CCL5. n=4 mice per genotype. Data are mean ± SEM., *P < 0.05, **P < 0.01 versus Apoe^-/-^ mice.

To determine whether the distinct CCR5 expression profile has functional consequences, we employed a chemotaxis assay on isolated splenocytes from Apoe^-/-^ and Apoe^-/-^ Mc1r^e/e^ mice and examined the migration of these cells towards the known ligands of CCR5 including CCL3, CCL4 and CCL5. Migration of Apoe^-/-^Mc1r^e/e^ CD4^+^ T cells towards CCL4 and CCL5 was significantly reduced (**Figure 6A**). Similar tendencies were also noted for Mc1r deficient CD8^+^ T cells (**Figure 6B**). Ly6C^high^ monocytes showed most drastic changes and CCL3-induced migration of these cells was particularly blunted in the Apoe^-/-^ Mc1r^e/e^ genotype (**Figure 6C**). Finally, to address whether the intracellular trafficking of CCR5 is compromised due to Mc1r deficiency, isolated and cultured splenocytes were stimulated with CCL5 followed by withdrawal of the ligand to evoke receptor recycling to the cell membrane. The experiment unveiled that CCR5 internalization as well as recycling was impaired in CD4^+^ T cells from Apoe^-/-^ Mc1r^e/e^ (**Figure 6D**). Likewise, CD8^+^ T cells from the same mice showed a lack of proper internalization and recycling response (**Figure 6E**). Overall, these results demonstrate a defect in CCR5 cell surface expression and recycling in the Apoe^-/-^Mc1r^e/e^ genotype, thus proving a possible explanation for the altered migratory behavior of T cells and monocytes.

## Discussion

The present study shows that MC1-R is intrinsically involved in the regulation of leukocyte production and migration, especially in conditions of excess dietary cholesterol. Hematopoietic MC1-R deficiency drastically increased tissue leukocyte counts in Apoe^-/-^ mice without affecting atherosclerosis. We also found that MC1-R is present in CD4^+^ T cells and the migration of these cells is disrupted by MC1-R deficiency.

In recent years, MC1-R has been recognized as a promising therapeutic target in various inflammatory diseases including atherosclerosis. Leukocytes, particularly monocytes and their descendant macrophages, contribute to the initiation and progression of atherosclerosis by accumulating in the subendothelial space and by destabilizing atherosclerotic lesions. Most of leukocyte subpopulations have been found to express MC-1R, which activation evokes a multitude of anti-inflammatory and pro-resolving responses (21). The evidence relies mainly on gain-of-function approaches that have utilized either the endogenous ligand α-MSH or more stable, synthetic agonists to address the therapeutic potential of targeting MC1-R for various inflammatory diseases. Owing to the multifaceted role of MC1-R in modulating inflammation, chronic activation of the receptor has been shown to provide protection against atherosclerosis in both pharmacological and genetic models (15, 26, 27). In macrophages, MC1-R has an additional regulatory function by promoting the clearance of excess intracellular cholesterol (15). This happens *via* induction of the ATP-binding cassette transporters ABCA1 and ABCG1, which initiate macrophage reverse cholesterol transport and prevent atherosclerosis. Although several studies have consolidated the favorable effects of MC1-R activation in leukocytes, little is known about the intrinsic role of MC1-R in these cells. Therefore, it remains largely an open question whether silencing of leukocyte MC1-R carries a reverse phenotype and leads to overproduction and accumulation of leukocyte subsets in tissues. To address this question, we employed bone marrow transplantation to generate a model of hematopoietic MC1-R deficiency and characterized the immunophenotype of this model in the context of atherosclerosis.

Strikingly, hematopoietic MC1-R deficiency led to a generalized increase of total leukocytes in the blood, bone marrow and spleen. This was mainly a result of expanded CD4^+^ T cell, B cell, monocyte and neutrophil populations and could have derived from induction of hematopoiesis. From a methodological standpoint, LSK^+^ cell count in the bone marrow was comparable between the genotypes indicating that hematopoietic MC1-R deficiency had not affected reconstitution efficiency following irradiation and transplantation. Likewise, the level of chimerism was comparable between the genotypes. Thus, the observed leukocyte profiles in Apoe^-/-^ Mc1r^e/e^ BM transplanted mice were not biased by a difference in BM reconstitution efficiency.

Of note, HFD-fed Apoe^-/-^ Mc1r^e/e^ chimeras showed increased frequency of total leukocytes, Ly6C^high^ monocytes and neutrophils in the bone marrow. These observations corroborate our earlier study on Apoe^-/-^ mice with global MC1-R deficiency (16). These mice displayed elevated total leukocyte and Ly6C^high^ monocyte counts in the bone marrow in response to HFD-induced hypercholesterolemia. Although we did not quantify stem cell count in that study, increased Ly6C^high^ monocyte frequency most likely reflects induction of hematopoiesis. It is well-established that hypercholesterolemia causes gradually developing monocytosis as a result of enhanced Ly6C^high^ monocyte production and increased survival of these cells in peripheral tissues (6). The development of monocytosis often requires elevation of plasma cholesterol concentration and this could have been the trigger for the monocytosis in Apoe^-/-^ mice with global MC1-R deficiency, which had elevated plasma cholesterol level after HFD feeding. However, in the present study, hematopoietic MC1-R deficiency caused monocytosis and enhanced HSC proliferation in the absence of exaggerated hypercholesterolemia. Some kind of an interaction between the genotype and cholesterol seem to exist since total leukocyte and HSC count were only increased in the bone marrow of HFD-fed Apoe^-/-^ Mc1r^e/e^ chimeric mice. Considering that cholesterol efflux pathways are disrupted due to MC1-R deficiency (15, 16), intracellular cholesterol levels might be accumulating in HSCs of Apoe^-/-^ Mc1r^e/e^ BM transplanted mice, rendering these cells more sensitive to hematopoiesis-triggering signals. Supporting this notion, we have previously observed reduced expression of ABCA1 and ABCG1 in the bone marrow of Apoe^-/-^ Mc1r^e/e^ mice (16), which could explain the hypersensitivity to HFD-induced hematopoiesis. Down-regulation of ABCA1 was also observed in this study along with reduced transcript levels of VCAM-1 and CXCL12, which are known to regulate proliferation and release of HSCs (35, 36). Collectively, these findings suggest that MC1-R is involved in HSC proliferation but it remains to be determined whether HSCs or nonhematopoietic cell types that form the HSC niche such as adipocytes, osteoblastic cells and endothelial cells express MC1-R and could MC1-R on those cells be functionally active and regulate hematopoiesis.

Exaggerated hypercholesteremia can induce myeloid-cell bias and lead to expansion of monocytes and neutrophils in the bone marrow and their release into the circulation (9). This occurs at the expense of suppressed lymphopoiesis, which could explain the present finding of increased bone marrow lymphocyte count in chow-fed Apoe^-/-^ Mc1r^e/e^ chimeric mice and the abolishment of this effect after HFD. Nevertheless, hematopoietic MC1-R deficiency was associated with significantly elevated lymphocyte counts in the circulation and spleen regardless of the dietary regimen. This was largely attributable to expansion of CD4^+^ T cells and B cells, which was accompanied by elevated plasma concentrations of different antibody subclasses as well as IL-2, IL-4 and IFN-γ. We also obtained evidence suggesting that the increase in CD4^+^ T cells is to some extent confined to pro-atherogenic Th1 effector cells. The principal Th1 cytokine IFN-γ and the transcription factor T-bet were significantly up-regulated in the spleen of Apoe^-/-^ Mc1r^e/e^ chimeric mice. Supporting the pro-atherogenic role of these markers, genetic deficiency of IFN-γ or T-bet attenuate lesion formation (42–44) while injection of recombinant IFN-γ accelerates atherosclerosis (45). Given this, combined with the fact that circulating monocyte numbers correlate with atherosclerosis progression, it was unexpected that the increased monocyte and CD4^+^ T cells did not aggravate atherosclerosis in Apoe^-/-^ Mc1r^e/e^ chimeric mice. On the other hand, global deficiency of MC1-R associated with larger and more vulnerable atherosclerotic plaques. However, this phenotype could have been primarily driven by increased plasma cholesterol level that arose as a consequence of disturbed bile acid metabolism (16). Hematopoietic MC1-R deficiency did not affect plasma cholesterol level, which thus allows to dissect the role of leukocyte MC1-R without confounding by a difference in cholesterol level. Even if elevated plasma cholesterol was the decisive factor for the observed phenotype in global MC1-R deficiency, increased numbers of leukocytes and particularly monocytes, neutrophils and CD4^+^ T cells in the circulation of Apoe^-/-^ Mc1r^e/e^ chimeras should have migrated into the atherosclerotic lesion and accelerated disease progression. The lack of effect on arterial leukocyte counts, lesional macrophage coverage and plaque size led us to investigate whether MC1-R deficiency influences the migratory behavior of leukocytes. *In vivo* homing assay revealed that CD4^+^ and CD8^+^ T cells as well as Ly6C^high^ monocytes from MC1-R deficient mice were preferentially entering the spleen. It is thought that T cells are trafficking between the spleen and inflamed atherosclerotic arteries through the circulation. Although we were unable to reliably track T cells that had entered the aortic wall, the reduced T cell count in the blood and paLNs suggest that MC1-R deficient cells are less likely to infiltrate aortic adventitia or atherosclerotic plaques.

When exploring the mechanistic explanation for the disturbed migratory behavior of leukocytes, we turned our attention to CD4^+^ T cells since previous studies have not provided conclusive evidence for the expression of MC1-R on these cells (25, 39). CD8^+^ T cells, B cells, monocytes and neutrophils, which were also increased in Apoe^-/-^ Mc1r^e/e^ BM transplanted mice, have been demonstrated to carry functional MC-1R (24, 25, 46, 47). To our knowledge, this is the first study to prove that MC1-R is present in CD4^+^ T cells, which opens the possibility that silencing leukocyte MC1-R signaling can directly affect this cell population as well.

We found that MC1-R deficient CD4^+^ T cells had up-regulated CCR5 mRNA and protein expression, while cell surface CCR5 level was reduced. Furthermore, CCR5-dependent cell migration and recycling of the receptor back to the cell surface after internalization were impaired in MC1-R deficient CD4^+^ T cells. Likewise, MC1-R deficiency drastically hampered the migration of Ly6C^high^ monocytes towards the CCR5 ligand CCL3. These findings imply that MC1-R deficient cells are upregulating CCR5 expression as a compensatory response to a defect in its recycling mechanism. CCR5 is expressed on various leukocytes subsets including monocytes and pro-inflammatory Th1 cells, where it mediates ligand-triggered (CCL3, CCL4 and CCL5) arrest and transendothelial migration (48). Genetic deletion or pharmacological inhibition of CCR5 reduces monocyte and T cell migration into lesions and protects against diet-induced atherosclerosis in Apoe^-/-^ mice (49, 50). Against this background, impairment of CCR5 function is a plausible explanation for the observed plaque phenotype in Apoe^-/-^ Mc1r^e/e^ chimeric mice that in the context of enhanced leukocytosis, would have otherwise showed signs of aggravated atherosclerosis. It remains to be still determined what is the exact mechanism behind disrupted CCR5 recycling. It is known that mutations in the MC1-R gene do not often block transcription or translation of the gene product but rather interfere with the intracellular traffic and lead to retention of the misfolded protein (51). Although it is purely speculative, MC1-R and CCR5 might be interacting and forming heteromers, which is a unique feature of GPCRs. Disturbed trafficking of mutated MC1-R might thereby also affect the internalization and recycling of CCR5.

In conclusion, the present study demonstrates that MC1-R is critically involved in the regulation of leukocyte production and migratory behavior. Hematopoietic MC1-R deficiency markedly increased tissue leukocyte counts and induced hematopoiesis in response to excess dietary cholesterol. However, CCR5-dependent migration was impaired in MC1-R deficient leukocytes. Opening a completely new avenue for investigation, we found that MC1-R is also present in CD4^+^ T cells, which are important for immune responses during host defense and play a central role also as drivers of autoimmune diseases and chronic inflammatory diseases such as atherosclerosis.

## Data Availability Statement

The raw data supporting the conclusions of this article will be made available by the authors, without undue reservation.

## Ethics Statement

The animal study was reviewed and approved by Animal Experiment Board in Finland, License Numbers: ESAVI/6280/04.10.07/2016 and ESAVI/1260/2020.

## Author Contributions

JK, ST, KT, AS, and PR performed the experiments. ST and MH developed methodology. JK and PR acquired and analyzed the data, prepared figures, and wrote the manuscript. PR designed and supervised the work. All authors contributed to the article and approved the submitted version.

## Funding

This work was financially supported by grants from the Academy of Finland (grant 316340 to MH and 315351 to PR), the Sigrid Jusélius Foundation (to MH and PR), the Finnish Cultural Foundation (to PR), Drug Research Doctoral Programme (to JK), the Finnish Foundation for Cardiovascular Research (to JK and PR), the Maud Kuistila Memorial Foundation (to JK) and Turku University Foundation (to JK).

## Conflict of Interest

The authors declare that the research was conducted in the absence of any commercial or financial relationships that could be construed as a potential conflict of interest.

## Publisher’s Note

All claims expressed in this article are solely those of the authors and do not necessarily represent those of their affiliated organizations, or those of the publisher, the editors and the reviewers. Any product that may be evaluated in this article, or claim that may be made by its manufacturer, is not guaranteed or endorsed by the publisher.
