# Supplementary material for: Melanocortin 1 Receptor Deficiency in Hematopoietic Cells Promotes the Expansion of Inflammatory Leukocytes in Atherosclerotic Mice
Source: Front Immunol. 2021 Nov 19;12:774013. doi: 10.3389/fimmu.2021.774013 (PMC8640177; doi:10.3389/fimmu.2021.774013)
Supplement: Supplementary file 1 [file DataSheet_1.pdf]

Supplementary Material

Supplementary Table 1. Quantitative RT-PCR primers for mouse genes.

**Gene Name**<br>Accession Number	**5'-3' primer sequence**
**ABCA1**<br>NM_013454.3	Forward: gcagatcaagcatcccaact<br>Reverse: ccagagaatgtttcattgtcca
**ABCA1**<br>NM_009593.2	Forward: gggtctgaactgccctacct<br>Reverse: tactcccctgatgccacttc
**ACTA2**<br>NM_007392.3	Forward: agattgtgcgcgacatcaaag<br>Reverse: gcagactccataccgataaagga
**ACTB**<br>NM_007393.5	Forward: tccatcatgaagtgtgacgt<br>Reverse: gagcaatgatcttgatcttca
**CCR1**<br>NM_009912.4	Forward: ggacaaaatactctggaaacacaga<br>Reverse: tgtgaaatctgaaatctccatcc
**CCR2**<br>NM_009915.2	Forward: agagagctgcagcaaaaagg<br>Reverse: ggaaagaggcagttgcaaag
**CCR5**<br>NM_009917.5	Forward: gagacatccgttccccctac<br>Reverse: gtcggaactgacccttgaaa
**CCR7**<br>NM_007719.2	Forward: tgtacgagtcggtgtgcttc<br>Reverse: ggtaggtatccgtcatggtcttg
**CCR9**<br>NM_001166625.1	Forward: caatctgggatgagcctaaacaac<br>Reverse: accaaaaaccaactgctgcg
**CD62L**<br>NM_011346.2	Forward: tgcagagagacccagcaag<br>Reverse: cagacccacagcttcaggat
**CXCL12**<br>NM_021704.3	Forward: tgcatcagtgacggtaaacca<br>Reverse: ttcttcagccgtgcaacaatc
**CXCR3**<br>NM_009910.3	Forward: taccttgaggttagtgaacgtca<br>Reverse: cgctctcgttttccccataatc
**CXCR4**<br>NM_001356509.1	Forward: gactggcatagtcggcaatg<br>Reverse: agaaggggagtgtgatgacaaa
**FOXP3**<br>NM_001199348.1	Forward: cccaggaaagacagcaacctt<br>Reverse: ttctcacaaccaggccacttg
**GATA3**<br>NM_008091.3	Forward: ctcggccattcgtacatgga<br>Reverse: ggatacctctgcaccgtagc
**ICAM1**<br>NM_010493.3	Forward: tggccctggtcaccgttgtgat<br>Reverse: aacagttcacctgcacggaccca
**IFNg**<br>NM_008337.4	Forward: tggctgtttctggctgttactg<br>Reverse: gctctgcaggattttcatgtca
**IL-4**<br>NM_021283.2	Forward: ggtctcaacccccagctagt<br>Reverse: gccgatgatctctctcaagt
**IL-17a**<br>NM_010552.3	Forward: tccctctgtgatctgggaag<br>Reverse: ctcgaccctgaaagtgaagg
**LFA-1**<br>NM_001253872.1	Forward: ccccagacttttgctactgg<br>Reverse: cgtgtgtccaggttgtagctc
**MC1R**<br>NM_008559.2	Forward: gtgctggttgtgatagccatc<br>Reverse: tgctgacacttaccatcaggt

**RORgt**<br>NM_011281.3	Forward: acagccactgcattcccagttt<br>Reverse: tctcggaaggacttgcagacat
**S1PR1**<br>NM_007901.5	Forward: atggtgtccactagcatccc<br>Reverse: cgatgttcaacttgcctgtgtag
**S29**<br>NM_009093.2	Forward: atgggtcaccagcagctcta<br>Reverse: agcctatgtccttcgcgtact
**T-BET**<br>NM_019507.2	Forward: gccagggaaccgcttatatg<br>Reverse: gacgatcatctgggtcacattgt
**TGFb**<br>NM_011577.2	Forward: ccgcaacaacgccatctatg<br>Reverse: cccgaatgtctgacgtattgaag
**VCAM1**<br>NM_011693.3	Forward: ggtcttgggagcctcaacggt<br>Reverse: agggccatggagtcaccgattt
**VLA4**<br>NM_010576.3	Forward: actccccacaggcctttatt<br>Reverse: tcagtcacttcgcagtttatttg

Supplementary Table 2. Body and tissue weights and plasma total cholesterol level in $Apoe^{-/-}$ and $Apoe^{-/-}$ $Mc1r^{e/e}$ chimeric mice on chow or HFD.

	**Chow**		**HFD**	
	**$Apoe^{-/-}$ → $Apoe^{-/-}$**	**$Apoe^{-/-}$ $Mc1r^{e/e}$ → $Apoe^{-/-}$**	**$Apoe^{-/-}$ → $Apoe^{-/-}$**	**$Apoe^{-/-}$ $Mc1r^{e/e}$ → $Apoe^{-/-}$**
**Body weight (g)**	29.0 ± 0.5	28.4 ± 0.4	28.3 ± 0.6	28.5 ± 0.9
**Spleen (mg)**	88.5 ± 5.3	97.7 ± 7.4	110.1 ± 3.6	139.3 ± 7.3***
**Gonadal WAT (mg)**	213.2 ± 26.0	267.0 ± 32.2	473.1 ± 43.2	404.1 ± 40.4
**Plasma total cholesterol (mg/dL)**	310.9 ± 21.0	305.3 ± 37.1	366.9 ± 12.3	364.7 ± 23.3

Data are mean ± SEM, ***$P < 0.001$ versus HFD-fed $Apoe^{-/-}$ mice. WAT indicates white adipose tissue

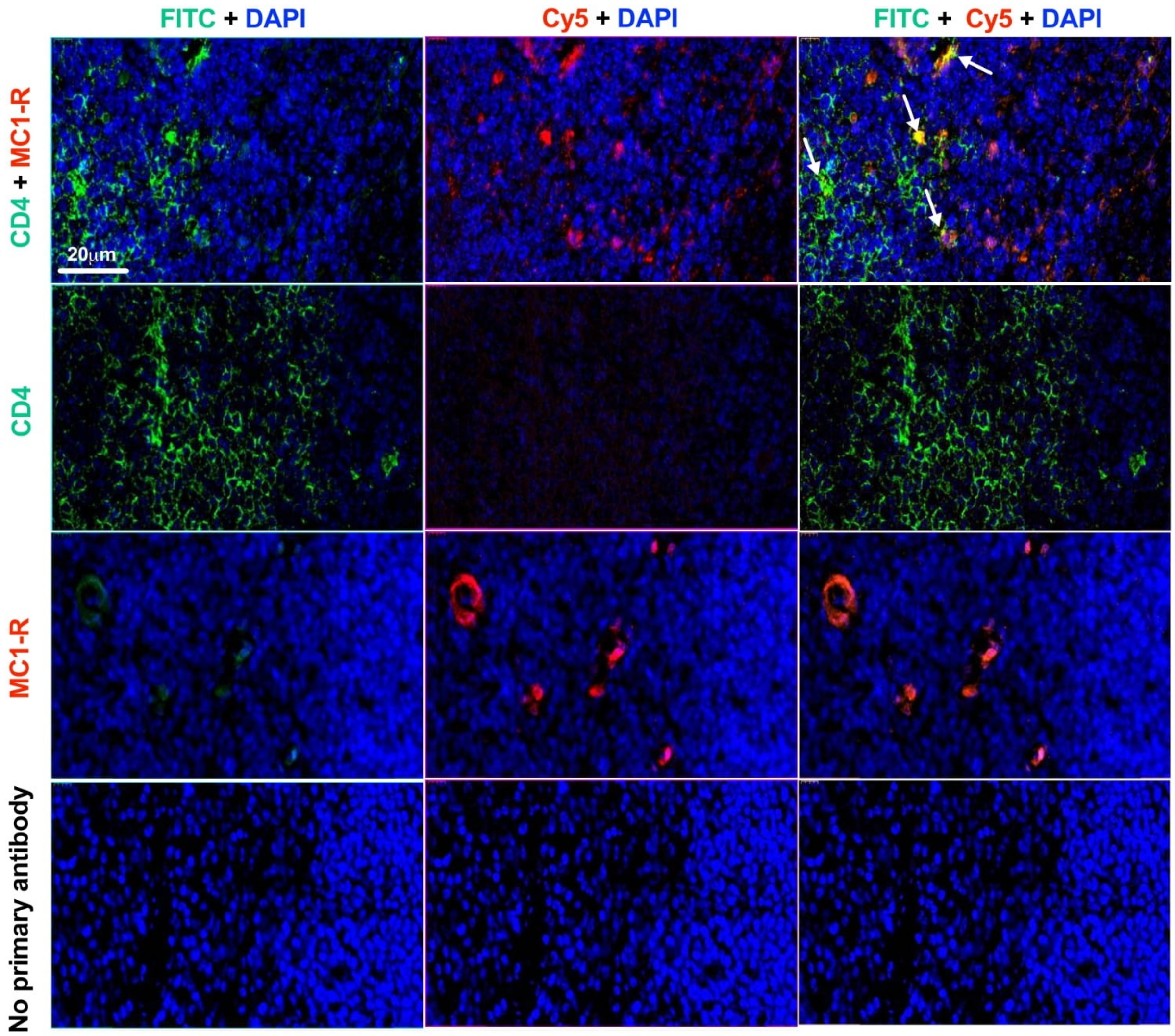

Supplementary Figure 1. Controls for MC1-R and CD4 immunofluorescence. Single stain controls were obtained by omitting either MC-R or CD4 primary antibody in the incubation. No primary antibody control was obtained by staining a consecutive section with the appropriate secondary antibodies only. Sections were otherwise treated and imaged in a similar fashion. Nuclei are visualized with DAPI in blue.

A

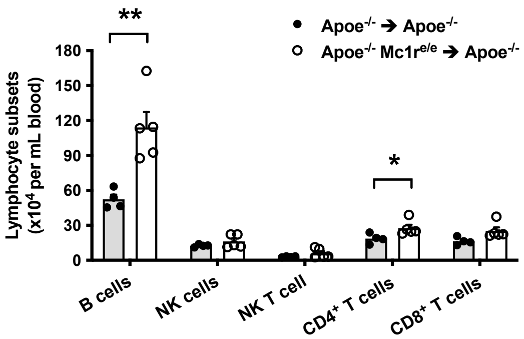

B

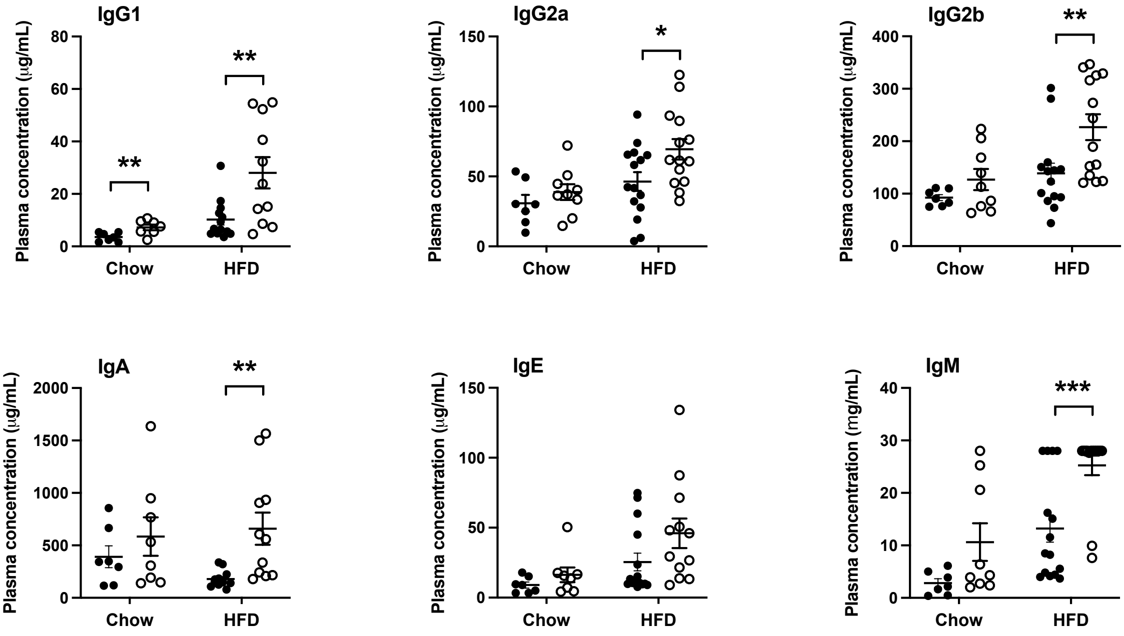

Supplementary Figure 2. Hematopoietic MC1-R deficiency increases circulating B and CD4^{+} T cells and plasma immunoglobulin levels. (**A**) Flow cytometric analysis of lymphocyte subsets in the blood of HFD-fed Apoe$^{-/-}$ and Apoe$^{-/-}$ Mc1r$^{e/e}$ chimeric mice. (**B**) Quantification of plasma immunoglobulin concentrations (µg/mL) in Apoe$^{-/-}$ and Apoe$^{-/-}$ Mc1r$^{e/e}$ chimeric mice. Data are mean ± SEM, *P < 0.05, **P < 0.01, ***P < 0.001 versus Apoe$^{-/-}$ mice. Each dot represents individual mouse.

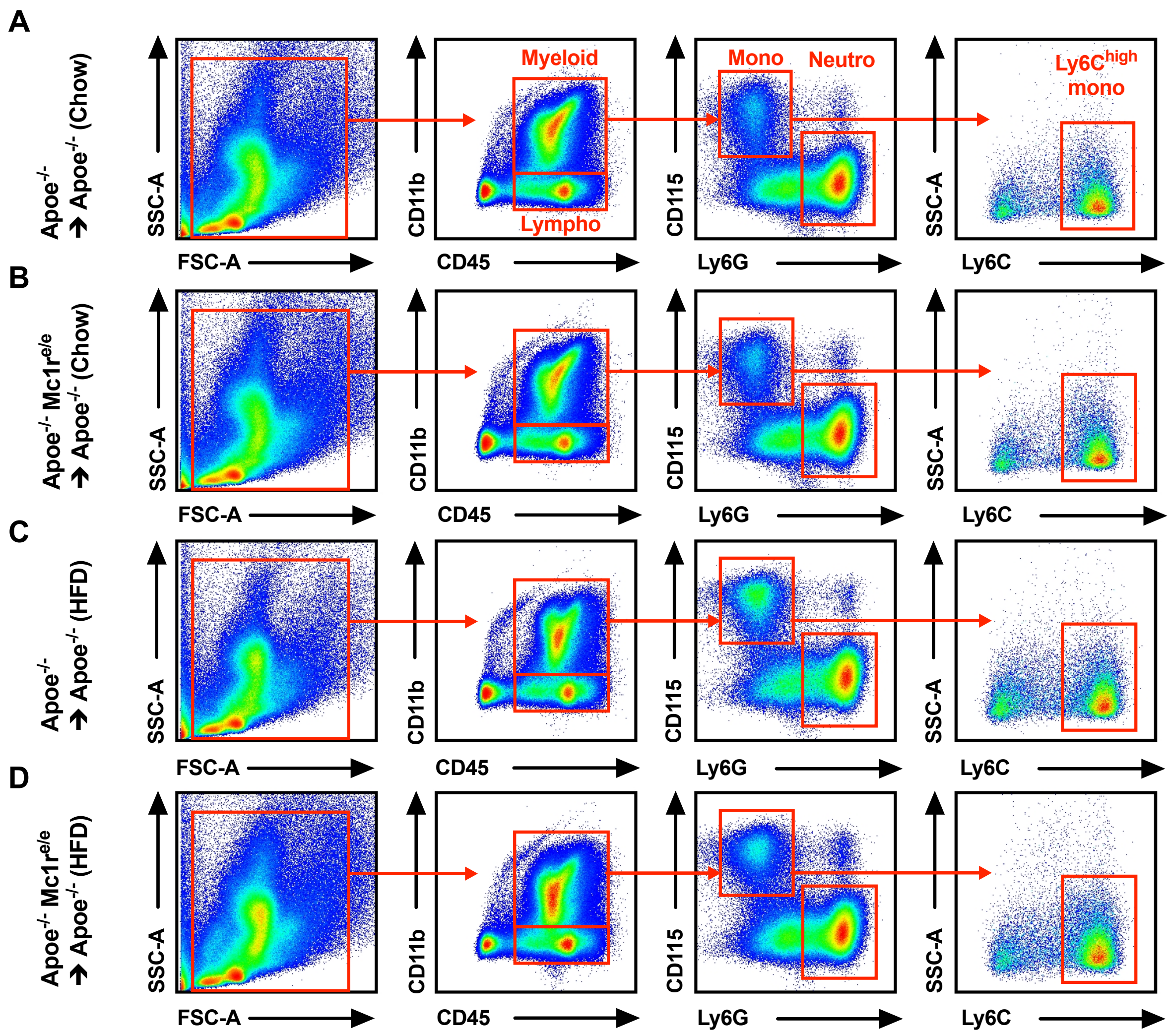

Supplementary Figure 3. Gating strategy for the quantification of leukocyte subpopulations in the bone marrow. (**A-D**) Representative dot plots for the gating of total leukocytes ($CD45^+$), lymphocytes ($CD45^+$, $CD11b^-$), neutrophils ($CD45^+$, $CD11b^+$, $CD115^-$ $Ly6G^+$) and $Ly6C^{high}$ monocytes ($CD45^+$, $CD11b^+$, $CD115^+$, $Ly6C^{high}$) in the bone marrow of $Apoe^{-/-}$ and $Apoe^{-/-}$ $Mc1r^{e/e}$ BM transplanted mice fed either a chow (**A** and **B**) or HFD (**C** and **D**).

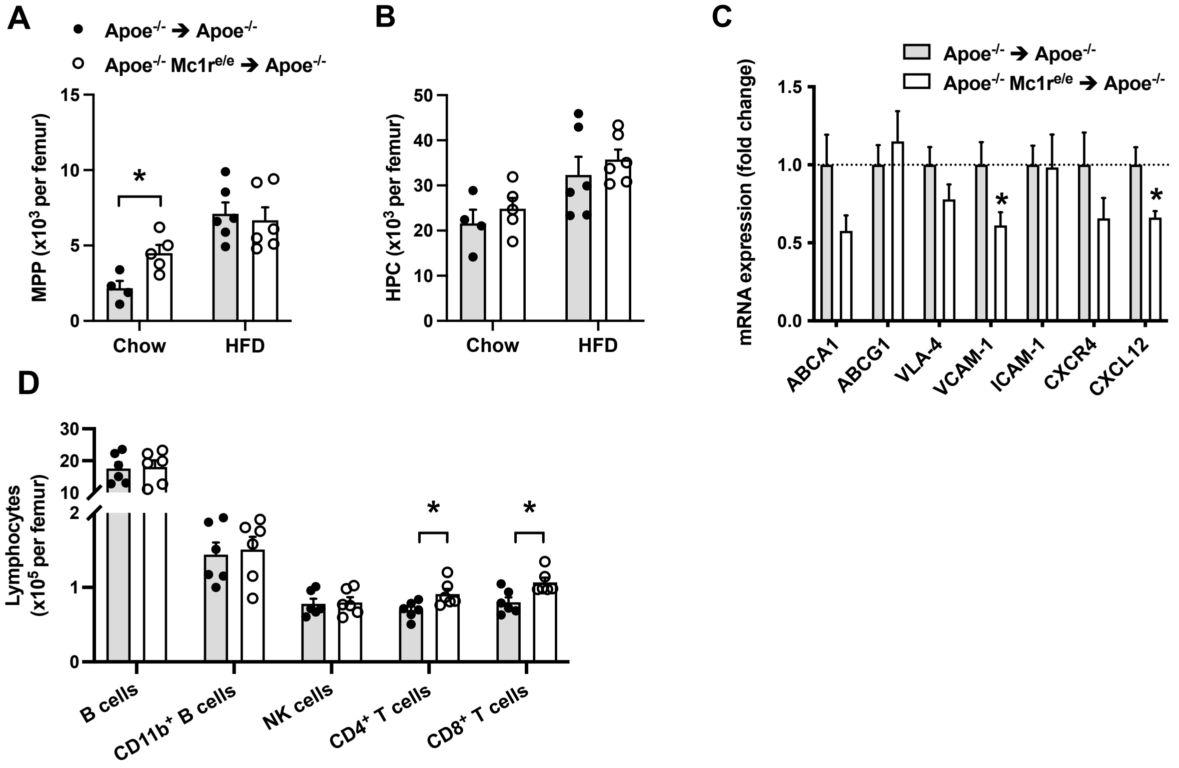

Supplementary Figure 4. Hematopoietic MC1-R deficiency enhances CD4^{+} and CD8^{+} T cells and multipotent progenitor cells in the bone marrow. (**A** and **B**) Quantification of multipotent progenitor cells (MPP) and hematopoietic progenitor cells (HPC) in the bone marrow of Apoe$^{-/-}$ and Apoe$^{-/-}$ Mc1r$^{e/e}$ mice. (**C**) Quantitative real-time PCR (qPCR) analysis of ATP-binding cassette transporter, chemokine receptor and adhesion molecule expression in the bone marrow of HFD-fed Apoe$^{-/-}$ and Apoe$^{-/-}$ Mc1r$^{e/e}$ chimeric mice. (**D**) Quantification of lymphocyte subsets by flow cytometry in chow-fed Apoe$^{-/-}$ and Apoe$^{-/-}$ Mc1r$^{e/e}$ chimeric mice. Data are mean ± SEM, *$P < 0.05$ versus Apoe$^{-/-}$ mice. Each dot represents individual mouse.

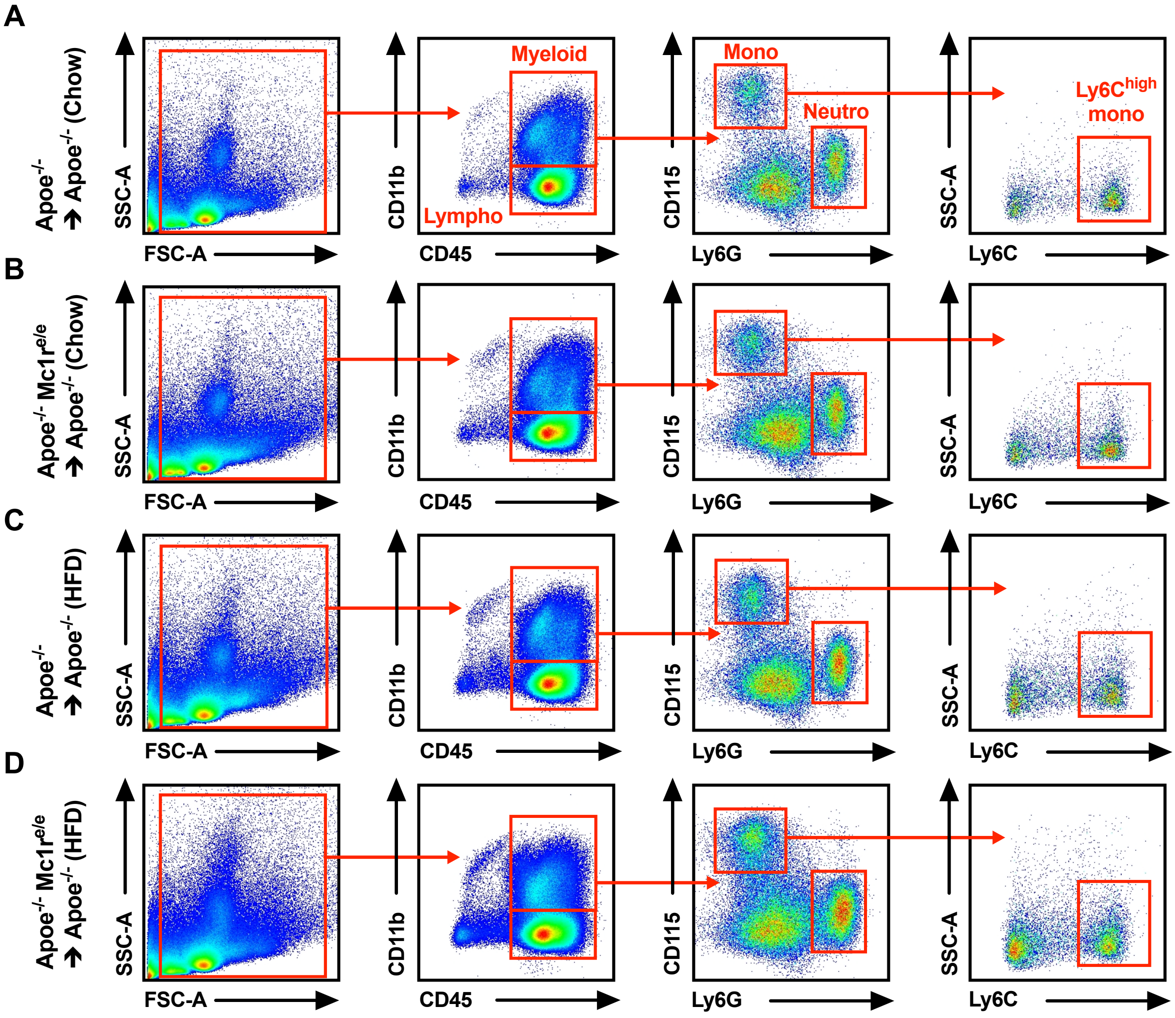

Supplementary Figure 5. Gating strategy for the quantification of leukocyte subpopulations in the spleen. (**A-D**) Representative dot plots for the gating of total leukocytes ($CD45^+$), lymphocytes ($CD45^+$, $CD11b^-$), neutrophils ($CD45^+$, $CD11b^+$, $CD115^-$ $Ly6G^+$) and $Ly6C^{high}$ monocytes ($CD45^+$, $CD11b^+$, $CD115^+$, $Ly6C^{high}$) in the spleen of $Apoe^{-/-}$ and $Apoe^{-/-}$ $Mc1r^{e/e}$ BM transplanted mice fed either a chow (**A** and **B**) or HFD (**C** and **D**).

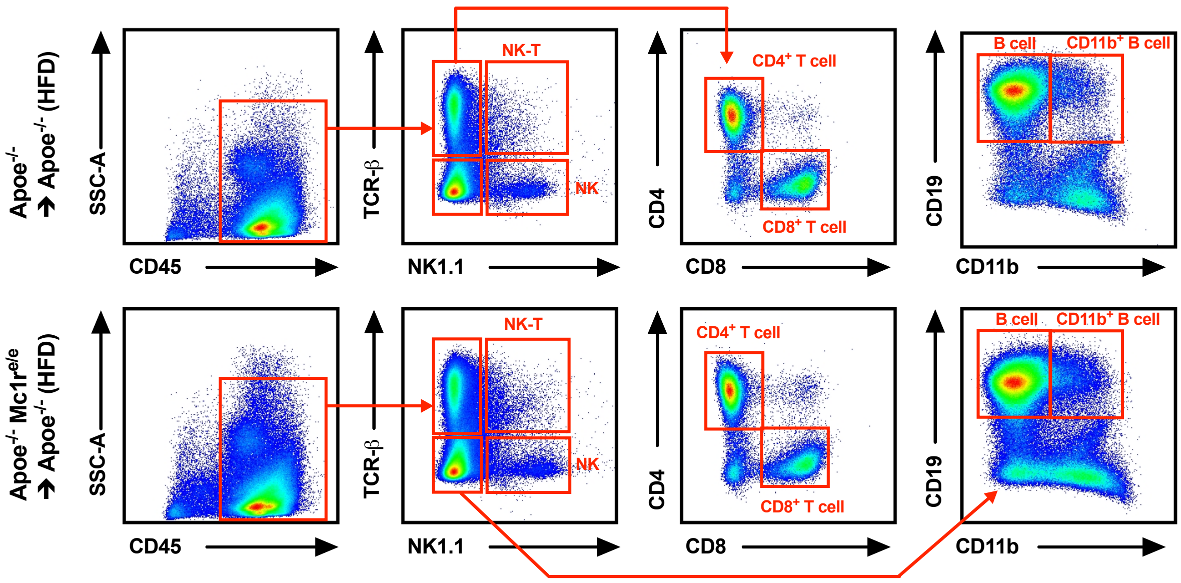

Supplementary Figure 6. Gating strategy for the quantification of lymphocyte subpopulations in the spleen. Representative dot plots for the gating of NK T cells (CD45$^+$, TCRβ$^+$, NK1.1$^+$), NK cells (CD45$^+$, TCRβ$^-$, NK1.1$^+$), CD4$^+$ T cells (CD45$^+$, TCRβ$^+$, CD4$^+$), CD8$^+$ T cells (CD45$^+$, TCRβ$^+$, CD8$^+$), B cells (CD45$^+$, TCRβ$^-$, CD19$^+$, CD11b$^-$) and CD11b$^+$ B cells (CD45$^+$, TCRβ$^-$, CD19$^+$, CD11b$^+$) in the spleen of Apoe$^{-/-}$ and Apoe$^{-/-}$ Mc1r$^{e/e}$ chimeric mice fed HFD.

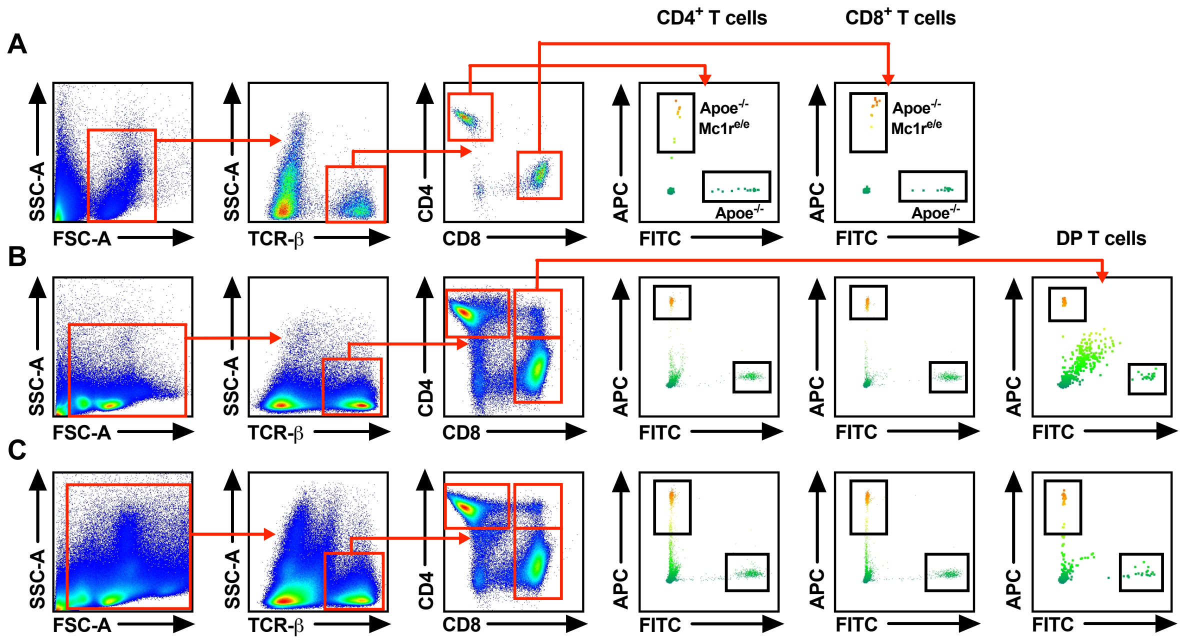

Supplementary Figure 7. Gating strategy for the identification and quantification of labelled T cells in the *in vivo* homing experiment. Splenocytes were isolated from Apoe$^{-/-}$ and Apoe$^{-/-}$ Mc1r$^{e/e}$ mice, labelled with CFSE or eFluor™ 670 and injected into recipient Apoe$^{-/-}$ mice. Twenty-four hours after the injection, CD4^{+} T cells, CD8^{+} T cells and CD4^{+} CD8^{+} double positive T cells (DP T cells) were identified by flow cytometry in the peripheral blood (**A**), peri-aortic lymph nodes (**B**) and spleen (**C**) of recipient mice. CFSE- (FITC channel) and eFluor™ 670 (APC channel) -labelled cells were quantified in each T cell subpopulation.

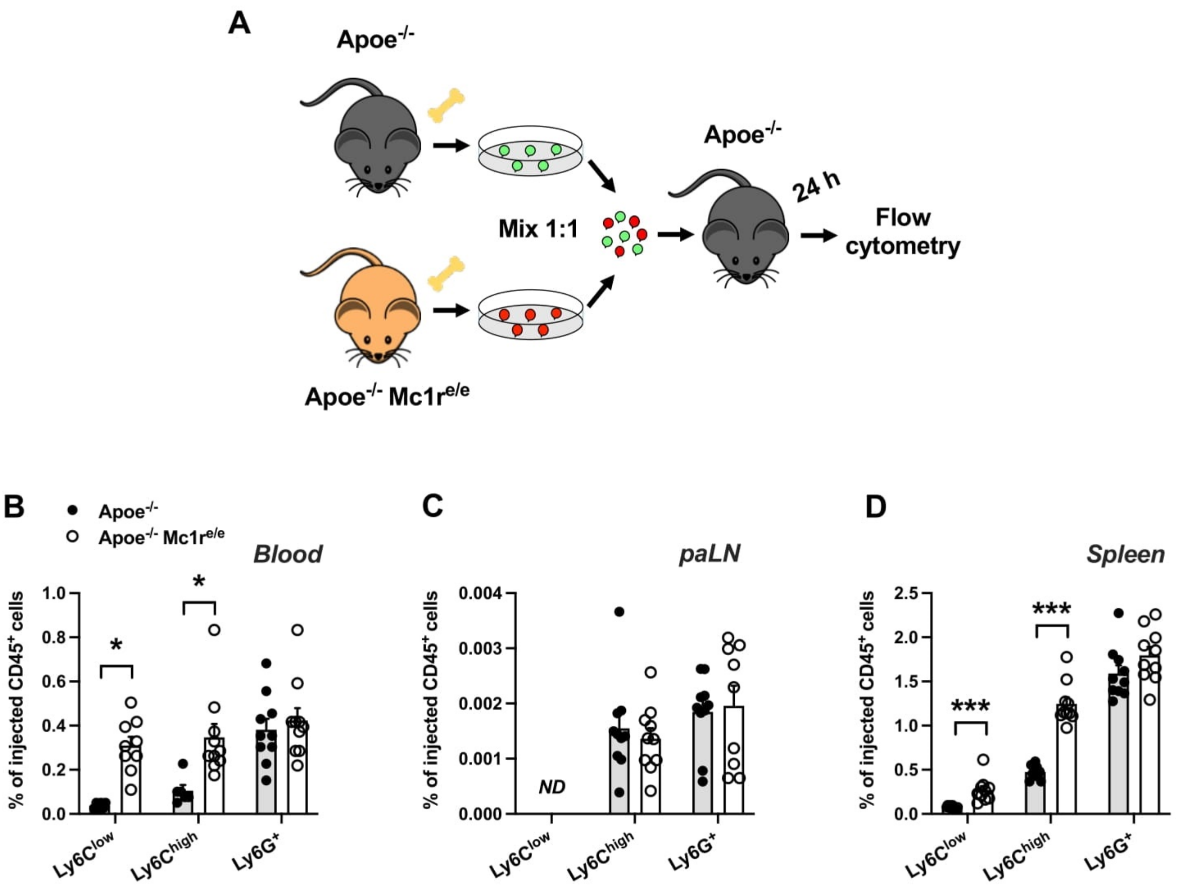

Supplementary Figure 8. MC1-R deficient monocytes preferentially home into the spleen. (**A**) Experimental design for analyzing homing of Ly6C^{high} and Ly6C^{low} monocytes and Ly6G^{+} neutrophils to the para-aortic lymph nodes (paLN) and spleen. Cells were isolated from the bone marrow of Apoe$^{-/-}$ and Apoe$^{-/-}$ Mc1r$^{e/e}$ mice and injected into recipient Apoe$^{-/-}$ mice. (**B-D**) Quantification of Ly6C^{low} monocytes (CD45^{+}, CD11b^{+}, CD115^{+}, Ly6C^{high}), Ly6C^{high} monocytes (CD45^{+}, CD11b^{+}, CD115^{+}, Ly6C^{low}) and neutrophils (CD45^{+}, CD11b^{+}, CD115^{-}, Ly6G^{+}) in the blood, paLNs and spleen as percentage of injected CD45^{+} cells. Data are mean ± SEM, *P < 0.05, ***P < 0.001 versus Apoe$^{-/-}$ mice. Each dot represents individual mouse.

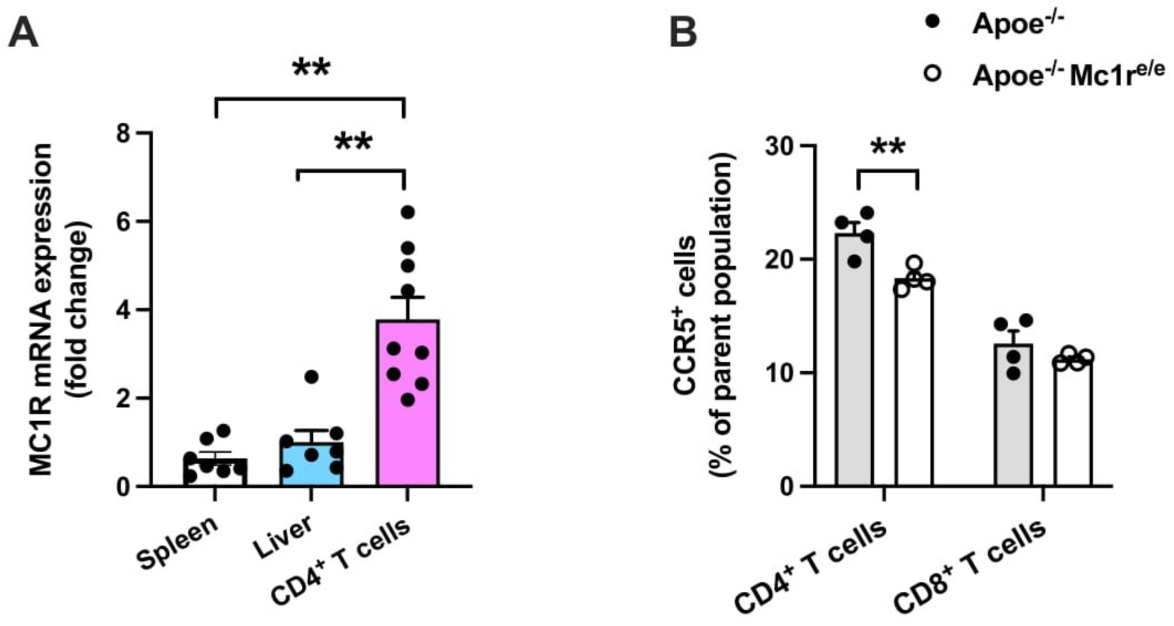

Supplementary Figure 9. (**A**) Quantitative real-time PCR (qPCR) analysis of *Mc1r* expression in the mouse spleen, liver and isolated splenic $CD4^+$ T cells. (**B**) Quantification of CCR5-positive T cells (expressed as percentage of $CD4^+$ or $CD8^+$ T cells) in the spleen of $Apoe^{-/-}$ and $Apoe^{-/-}$ $Mc1r^{e/e}$ mice. Data are mean ± SEM, **P < 0.01. Each dot represents individual mouse.
